# Macroeconomic dynamics in a finite world based on thermodynamic potential

**DOI:** 10.1038/s41598-023-44699-y

**Published:** 2023-10-21

**Authors:** Éric Herbert, Gaël Giraud, Aurélie Louis-Napoléon, Christophe Goupil

**Affiliations:** 1 Université Paris Cité, CNRS, UMR 8236-LIED, 75013 Paris, France; 2 Environmental Justice Program, McCourt School of Public Policy, Georgetown University, Washington, DC USA; 3 Chaire Énergie et Prospérité, Paris, France

**Keywords:** Energy science and technology, Physics

## Abstract

This paper presents a conceptual model describing the medium and long term co-evolution of natural and socio-economic subsystems of Earth. An economy is viewed as an out-of-equilibrium dissipative structure that can only be maintained with a flow of energy and matter. The distinctive approach emphasized here consists in capturing the economic impact of natural ecosystems’ depletion by human activities via a pinch of thermodynamic potentials. This viewpoint allows: (*i*) the full-blown integration of a limited quantity of primary resources into a non-linear macrodynamics that is stock-flow consistent both in terms of matter-energy and economic transactions; (*ii*) the inclusion of natural and forced recycling; (*iii*) the inclusion of a friction term which reflects the impossibility to produce (and recycle)goods and services without exuding energy and matter wastes, and (*iv*) the computation of the anthropically produced entropy as a function of metabolizing intensity and frictions. Analysis and numerical computations confirm the role played by intensity and frictions as key factors for sustainability by contrast with real gdp growth—as well as the interplay between resource scarcity, income inequality, and inflation. A more egalitarian society with moderate inflation turns out to be more sustainable than an unequal society with low inflation. Our approach is flexible enough to allow for various economic models to be embedded into our thermodynamic framework. Finally, we propose the open source EcoDyco software as a first complete realization implementing economic dynamics in a multi-resource environment.

## Introduction

A prominent stream of economic literature considers Nature as an easy-to-overcome constraint for economic activities. Unlimited supply of energy and matter (hereafter E &M) and full—or, at least, partial—substitutability of production factors—to wit, labor *L*, capital *K*, matter, *M*, or energy *E*—are often assumed (see OFCE^1^ and Nordhaus^2^). These modeling choices have been questioned in the environmental context of a finite and hotter planet (see Meadows et al.^3^, Fankhauser et al.^4^, Giraud^5^ and Stern et al.^6^). The consequences of direct and incidental environmental damages induced by global warming (IPCC^7^) biodiversity erosion (IPBES^8^) and a growing resource scarcity (e.g., fossil fuels, metals, or rare earth elements, IRP^9^) are so severe that a cogent economic modeling can not longer ignore them. On the other hand, Material Flow Analysis (MFA hereafter) has now amply demonstrated that flows of E &M deeply shape our societies (see Fischer-Kowalski^10,11^) while Fisher-Kowalski^12^ suggests that changes in a society’s energy regime may even foster political revolutions. This literature provides us, today, with a wealth of data to better understand the climate/biodiversity/resources nexus and its interplay with the human sphere. An abiding analytical challenge, though, is to provide a unified framework within which we can start to make sense of the complexity of these interrelationships. The aim of this paper is to offer such a set-up by embedding a simple economic dynamics within a thermodynamic setting.

As early as in the first half of the 20th century, indeed, thermodynamics has been viewed as the natural framework where one should address the influence of E &M and ecosystems on the anthroposphere: *“The laws [of thermodynamics] that express the relation between matter and energy, govern the rise and fall of political systems, the freedom or bondage of societies, the movements of commerce and industries, the origin of wealth and poverty, and the general physical welfare of people.”* (Frederick Soddy^13^).

In the wake of Georgescu-Roegen, many scholars have plead in favor of integrating economics and thermodynamics, see, e.g. Georgescu-Roegen^14^, Ayres^15^, Ayres^16^, Berg^17^, Kummel^18^, Dafermos^19^, and Galbraith and Chen^20^ Baumgärtner^21^ mobilizes the joint-production concept in order to account for the production of unwanted outputs in addition to manufactured goods. According to Söllner^22^, coupling thermodynamics and economics can be attempted at various levels: (*i*) by introducing an energy theory of value, see, e.g., Odum^23,24^; (*ii*) through various analogies and metaphors like, for instance, in Samuelson and Saslow ^25,26^ and Sousa^27^ (*iii*) or, else, by adding thermodynamic constraints to a stylized economy as, e.g., in Ruth, Georgescu-Roegen and Boulding^28–30^ and Mayumi^31^.

This paper belongs to the third approach. Indeed, on the one hand, we don’t claim to provide a foundational theory of value, on the other, analogies often run the risk of leading to improper applications of thermodynamics. Instead, we provide a rigorous non-equilibrium thermodynamic setting where the first two principles of thermodynamics are fulfilled, and we analyze their consequences on a phenomenological economic dynamics. At variance with part of the literature in ecological modeling based on thermodynamics, we refrain from relying on additional principles beyond the first two laws, such as the so-called maximum power principle (mpp)—see Odum, Hall and Goupil^23,32,33^. Moreover, we quantitatively decipher the consequences of natural resources and absorption capacities of wastes being both finite. Thus, our thermodynamic viewpoint also fulfills the requirement formulated by Bestiaire^34^, namely, while casting an economy into its environment, to describe its interactions with the physical limits of its surrounding.

Our resulting (continuous-time) dynamics typically exhibits the type of dynamic complexity described by Sterman^35^, Table 2, as characteristic of modern-world problems: with the exception of the quantity of E &M (first law), no variable remains invariant across time; the biophysical and the anthropic spheres are tightly coupled and the feedback from one sphere onto the other is a key driver of our overall scenarios; their dynamics is non-linear and path-dependent; both spheres are self-organizing, open, nonequilibrated dissipative structures that acquire, store and utilize E &M, adjusting their metabolism to the other sphere’s behavior; delays in feedbacks imply that counterintuitive trade-offs may emerge: temporarily going beyond the regeneration speed of the biosphere does *not* lead to an immediate breakdown but the sustained exceeding of carrying capacity ends up causing economic extinction, Hence, the need to slow down the economy without necessarily embarking into degrowth. Finally, howbeit we assume neither a maximum nor a minimum entropy principle, the E &M-driven economic metabolism emerges as an “island of order” within its increasingly disordered surrounding—much in line with “culture” being sometimes described as a quest to store more energy^36^. We are then able to compute the export of entropy by the anthroposphere to the biosphere as a function of the characteristics of the economic metabolism.

From an economic viewpoint, we add to the literature devoted to the celebrated controversy between Nicholas Georgescu-Roegen and Herman Daly versus Robert Solow and Joseph Stiglitz, see Daly^37,38,39^. Solow^40^ went as far as to write: “[t]he world can in effect get along without natural resources” . This paper provides a thermodynamically sound and transparent framework highlighting the opposite statement which insists on the intrinsic incapacity of a human economy to produce anything without borrowing E &M from its surrounding. It therefores pays tribute to the literature which, at least since Jevons^41^, alerts on the limitation of natural resources. However, taking into account the natural recycling and regeneration of a (limited) number of resources, we do not conclude that economic growth *per se* is unsustainable: rather, we conclude that the speed at which Nature regenerates (which ultimately depends solar energy input) imposes an upper-bound on economic growth—even when recycling is taken into account, as recycling *also* requires additional energy. This is what Japan^42^ seems to have understood in the 18th century: a demand for woods that would inflate at a speed exceeding the one at which trees grow would have led to a catastrophic collapse of a deforested archipelago. The Tokugawa era might have been one of the few periods in modern times where a global human society deliberately chose to slow down its economy in order to adjust to natural cycles. This paper argues that this lesson holds for every type of economy.

Already since the beginning of the Georgescu-Roegen/Solow controversy, technological optimists have advocated that technological innovation might release the constraint of finite natural resources. In 1997 Robert Ayres^43^ thus wrote: “there is no definite upper-limit [of production] given the possibility of dematerialization, re-use, renovation, recovery and recycling”. A usual objection is the famous Jevons’ paradox^44^ (or rebound effect): heretofore, technological progress never demonstrably reduced aggregate demand for energy. We feel quite compelled by Jevon’s criticism. This paper, however, evidences that, *even if we concede* the possibility of an exogenous technological progress whose effect is not canceled by some^45^ “intrinsic human addiction to the comfort offered of exosomatic instruments”, there is an upper-limit on the intensity at which an economic dissipative structure can metabolize natural resources without “dying” in the medium-run. This, however, does not imply that moderate growth is impossible, provided its rate vanishes in the long run.

Perhaps the unique attempt to introduce E &M conservation as well as the entropy law in a formal accounting set-up can be found in Krysiak^46^ (see also Couix^47^). Our paper fits into the formal setting introduced there: We confirm that unbounded growth is impossible and that weak sustainability is unfeasible. As Krysiak writes, however, “[his] results indicate only that limits to growth for the production of most physical goods are likely to exist, they do not quantify these limits and thus do not imply that such limits will be met in the foreseeable future.” We provide a full-blown characterization of such limits both in terms of physical finiteness constraints and of entropy production.

The remainder of this paper is organized as follows. The next section informally describes the main characteristics of our model. “Thermodynamics and the energy conversion engine” section recalls some basics of out-of-equilibrium thermodynamics for the non-specialist reader. In “Conversion engine in an economic context” section, we describe our physical sheet, built with thermodynamics-based categories, and we examine various examples in “Resource case studies” section. Finally, “The anthroposphere” section embeds an sfc-economic dynamics into our physical sheet and shows the effects of limited resources, friction, and intensity on the economic macrodynamics.

### Specific features of our thermo-economic approach

Let us first describe the specific features of our model (a short summary is available in Table 1):

1. Any sensible modeling of an economic dynamics must not only expatiate the dependence of production and consumption upon E &M, but also expound a frictional or viscosity term, responsible for neither energy nor matter being entirely used up during the metabolic process. Instead, a fraction of them is dispersed as waste. Thermodynamics dictates that the influence of the frictional or viscous term depend on the operating intensity of the metabolic process, just as it does for any physical system. In other words, to produce “cleanly” is to produce slowly, the reverse assertion being false in general.
2. The economic sphere exchanges energy and matter with the biosphere via an aggregate *demand function* for inputs which plays a role analogous to the traditional economic * aggregate production function*—the difference being that, more often than not, E &M are not even explicit inputs of this production function. They are roughly similar only in the case of unlimited resources. However, even in this case, the empirical accounting of aggregate production functions is plagued by well-known methodological issues, while our material demand function is backed by a significant fraction of the MFA literature. In addition, here, we view an economy as a large dissipative structure^48^, so that production is characterized by a friction term that defines the amount of rubbish produced at a given metabolizing intensity. As a proxy for the inverse of technological progress, this friction is subject to exponential growth due to erosion, or decay in the case of investment and technological progress. The lower the friction term, the easier it is to collect and transform material resources. This fluidization of the extraction/production process may then naturally lead to accelerated depletion of resources insofar as the operating intensity of the economic metabolism is now able to climb. Conversely, high friction necessarily imposes a low metabolizing intensity, and therefore, as we shall see, a low gdp growth.
3. A modeling structure based on elementary unit, each simulating a single resource, called physical sheet in the following. Every physical sheet inner structure shares features of a conversion engine. The physical world is made up of different core-linked sheets, which guarantees a limited growth in modeling complexity proportional to the number of sheets.
4. The formalism of the elementary sheet is that of a conversion engine, which embodies some of the categories used in finite time thermodynamics. Intensity—i.e., the speed at which the economy operates—and friction characterize the state of the productive infrastructures: an economy with a poorly functioning production framework involves a lot of “frictions” in its operations.
5. The conservation of energy and matter requires that both E &M be either in the primary source, in the waste reservoir, or in end-use products. For each resource, the sheet structure is formally identical and one sheet corresponds to a specific resource. The use of several sheets makes it easy to account for several resources. Thus, even if the elementary structure is simple, making the model more complex by increasing the number of elementary sheets is not a problem.
6. For the sake of concreteness, we have chosen to associate a single sheet—for respectively energy and matter (see additional information in section Appendix for a complete multi-resources case study)—to a stock-flow coherent (sfc) setting—see^49^—and a Goodwin-Keen^50^ type of dynamics. For simplicity, the economic sphere is composed only of three sectors: households, firms and banks. We use the empirically calibrated version for the world economy which has been successfully applied by^51–53^ and^54^ for the more specific study of climate change. At any time, the existing stock of installed capital, when used at full capacity, dictates a certain level of metabolizing intensity (provided there are enough labor forces). The metabolizing process, in turn, requires natural resources to be extracted and depleted at a corresponding speed. The availability of resources (captured through the high entropy potential) and the carrying capacity of Earth as a sink for anthropic junk (captured though the low entropy potential) enable the metabolizing intensity to materialize into an aggregate “useful work”—the output of the whole economic process—associated with wastes. Part of this work leads to final commodities and services which are consumed by households. Another fraction serves as investment in new capital. Meanwhile, installed capital decays. Both consumption and capital depreciation fuel another flow of scums which feeds the low-entropy potential. The modified installed capital induces in turn a change in the metabolizing intensity, etc.

Our resulting dynamics is not only sfc in the macroeconomic sense, but also in the resource-flow sense: it conforms to the conservation of matter and energy (first law). On the other hand, it also fulfills a basic “correspondence principle” : when natural resources are infinite, our framework boils down to a standard macroeconomic model *à la* Akerlof-Stiglitz^50,55^. Of course, when resources are finite, the interplay between the physical sphere and the anthropic one sharply modifies the dynamics. In particular, an economy entirely based on non-renewable energies and matter must end up in finite time in a state of (nearly) zero activity.

Our results are worth being compared with^56^. There, three scenarios are simulated for the French economy: (a) The green growth scenario, based on technological progress and environmental policies, achieves a significant reduction in ghg at the cost of increasing income inequality and unemployment; (b) The policies for social equity scenario adds direct labour market interventions that result in an environmental performance similar to a) while improving social conditions; (c) The degrowth scenario further adds a reduction in consumption and exports, and achieves a greater reduction in emissions and inequality with higher public deficit. Here, by making the finite resource constraint explicit, we show that, even with exogenous technological progress, the green growth scenario is unfeasible in the long run at the world level. However, we do not conclude that degrowth is the unique (strongly) sustainable option: reducing inequality by increasing wages, decreasing the profit share, and tolerating some inflation enables to find out a path similar to b) provided the yearly output be upper-bounded across time.

**Table 1:** Summary of the model’s features.

1—Quantity and quality	Manufacturing of material objects requires a nonzero quantity of energy of high quality to reduce and reverse the dispersion process of matter
2—Economic/resource spheres	The availability of resources and the carrying capacity of Earth as a sink for anthropogenic wastes enable the production of aggregate “useful work”-the output of the whole economic process-associated with wastes
3—Frictional viscous term	Joint production is composed of garbage and work, whose respective fractions depend on the operating intensity, and is irreversible
4—Aggregate demand function	Global demand is disaggregated to quantify the demand for each resource. It boils down to the standard “production function” in case of unlimited resources
5—Common elementary unit	Each resource (matter and energy) is described by the same structure, called a *sheet*
6—Energy conversion engine	The formalism of each sheet is based on Finite Time Thermodynamics, characterized, among other things, by friction and operating intensity
7—E&M conservation	The quantity of each physical resource is conserved throughout the economic process. The economic dynamics is Stock Flow Consistent. Each sheet satisfies both physical and economic balance

## Thermodynamics and the energy conversion engine

This section specifies some thermodynamic concepts used in the proposed modeling.

### First and second laws

The first law of thermodynamics states that energy is conserved, which means that it can neither be created nor destroyed. This 1st law is equally applicable to matter in the sense that, in a closed system, matter is conserved. This naturally applies to the Earth^22^: the respective total amounts of carbon, copper, iron, rare earth elements... cannot change significantly over time. On the other hand, the Earth is not an isolated system since it receives a very large flow of energy from the Sun. The second principle states that, even if the total amount of matter is constant, its quality can change over time and thermodynamics essentially describes the transformations from one quality to another^31^. During so-called spontaneous transformations, energy changes quality from a concentrated form, called work, to a diluted form, called heat. The same law affects matter, which, over time, spontaneously degrades, also passing from a concentrated form to a diluted one. This observation does not justify invoking a new principle of thermodynamics, contrary to what has been sometimes advocated. Indeed, changes in the quality of matter are already included in the second law. The production processes of consumption and capital goods make use of matter that can be described as concentrated, compared to what happens to this matter when it is then diluted in the trash we generate during both operations.

Recycling matter is therefore in itself a procedure of concentrating the matter to make it usable again as a resource. For each element and matter resource, we define a potential characterizing its more or less concentrated state. In the case of mineral resources, the concentrated form corresponds to the matter concentrated in the mine, while the diluted form refers to the matter dispersed in the Earth’s crust, oceans, or atmosphere. As a result, the energy cost to reconcentrate a diluted species is perfectly calculable and used in ecological economics; see^57,58^.

### Intensive, extensive property

Another thermodynamic concept is the distinction between extensive and intensive properties. An extensive property depends on the quantity of matter in the system: when dividing a system in two equal parts, each half is endowed with half the amount of the considered property (like mass or volume). An intensive property is independent of the amount of matter; see^59^. Examples of intensive properties are temperature, pressure, and chemical and electrochemical potentials. As they serve to define thermodynamic potentials, intenstive properties are essential for the mathematical formulation of the second principle and its application in terms of exergy.

### Thermodynamics conversion and boundary conditions

Living systems, such as organisms and ecosystems, are physical systems, working under non equilibrium conditions. They constitute a specific part of the large family of the thermodynamic systems, of which the Carnot engine is the most famous. In the spirit of^60–63^, and other authors, we shall consider the physical world as a thermodynamic conversion system, which, like a living organism, metabolizes E &M.

In the economics literature^30^, introduced the distinction between “cowboy” and “spaceman” viewpoints. In the former, the Earth possesses unlimited reservoirs of E &M: production and consumption are never considered as physical constraints, usually because technology, monetary capital, or labor are postulated to be substitutable to environmental resources. For a “spaceman” economy, reservoirs are limited, as well as waste capacity, and no human activity can overcome these limitations. Our approach is in the “spaceman” spirit. We insist that the biosphere does not exchange matter with the rest of the universe, but only energy. Production and consumption flows of matter are hence limited in quantities. However, qualities can be improved if there is a sufficient high-quality energy supply. The same idea can be found in^31^: “... energy which is sourced from a stock, like oil, can only be used once. Energy which is sourced from a flow, like solar or geothermal energy, cannot be used at a rate exceeding the source flowrate.” Before the late 18th century, economies were organic^64^: societies were depending on the annual amount of photosynthesis conversion in plants. Economies were hence “flow economies,” limited by the solar energy flow. Since the Industrial Revolution, economies have been mostly based on fossil fuels, and have been depended on their available stock: they are “stock economies.” As a consequence, the nature of the boundary conditions at the edges of the human sphere—flow vs. stock conditions—leads to very different outcomes for an economy. Under flow conditions, divergence towards an economic metabolism operating at an ever-increasing intensity is impossible because it is limited by the flow of energy supply. Under stock conditions, any improvement in the conversion process is made possible by the energy stock (until it is depleted). As a result, under stock conditions, the trend towards continuous growth in conversion intensities becomes possible. This is, in short, our current situation, and makes it necessary to consider how the finite size of resources forces and enables us to bifurcate from this trajectory.

Thermodynamics principles have two implications in an economy^22,31,65^. First, if E &M are both coming from a stock (e.g., the respective mass of matters in the Earth, the energy contained in fossils fuels), with no recycling, they both can be exhausted. The energy coming from the Sun does not stem from a (human-sized) stock, but is limited by the total solar energy flux the Earth receives. Consequently, the ability of the human sphere to divert energy from biomass and oceans is limited. Second, since neither matter, nor energy can be destroyed, the resulting debris has to appear somewhere. Hence, thermodynamics constrains economics: (*i*) by input availability and (*ii*) by output sink capacity. This means that (*i*) not only the (non conventional) peak oil or the extraction peak of copper—see^66^—will have a huge impact on the capacity of the world economy to keep increasing its gdp but also (*ii*) climate change and biodiversity erosion, which can both be viewed as symptoms of the limited capacity of environmental sinks to absorb our wastes.

### Efficiency and production

**Figure 1:**
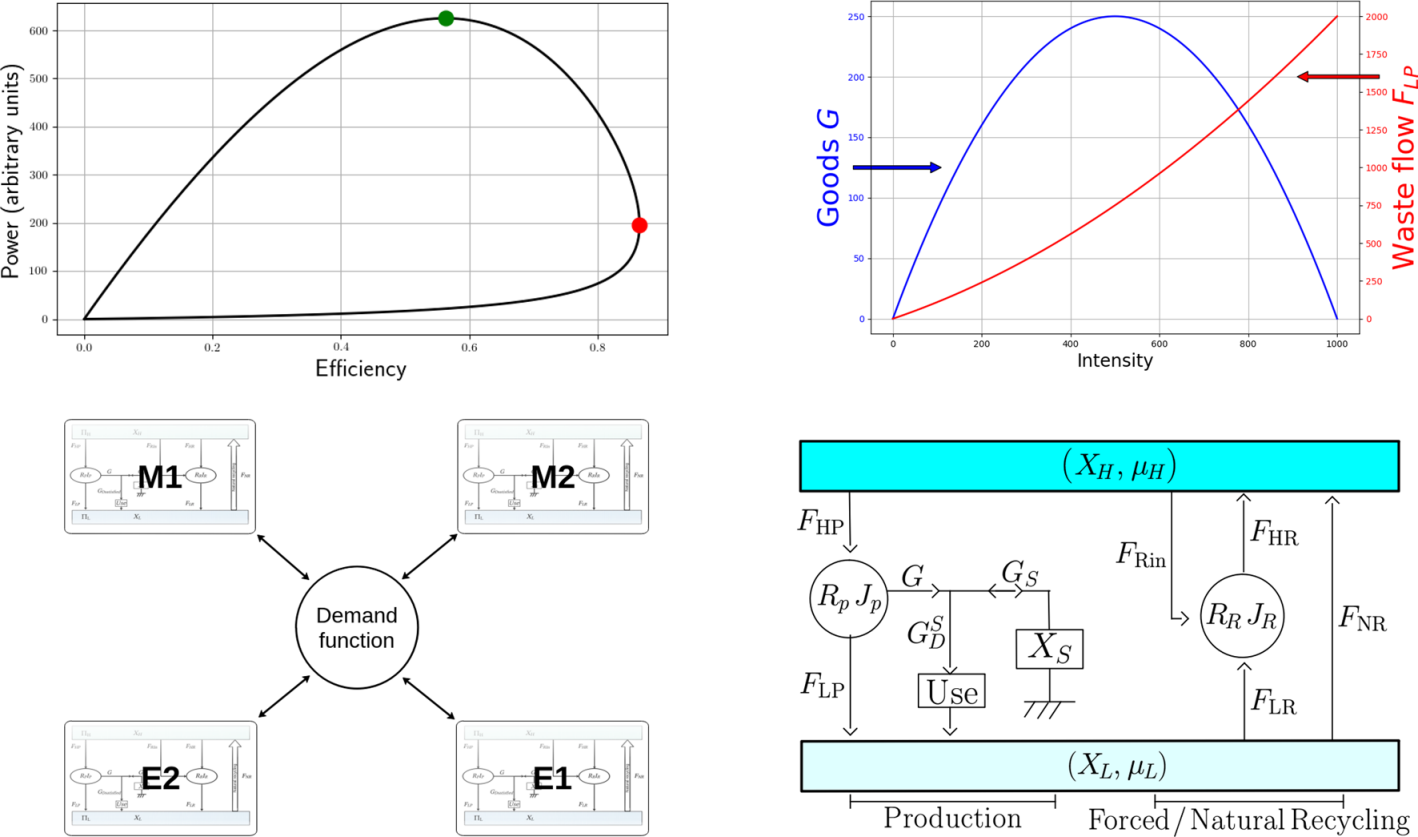
Captions correspond to figures in the normal reading direction. (**a**) Power in function of efficiency of an energy conversion engine^67^ at constant heat sources temperature, i.e. $\Delta \mu$ is constant. Increasing the working intensity from 0, maximum efficiency is reached first (red circle) and then the maximum power (green circle). (**b**) Goods and waste flow over intensity, with $R=10^{-3}$ and $\Delta \mu =1$. (**c**) Physical sheets with four sectors interconnected by a central kernel. (**d**) Global structure of a sheet of resource. Left part is the production area, middle part is forced recycling, and right part is natural recycling.

Consider now a conversion engine able to convert the resources E &M into goods and services, *G*. We shall consider that the potential of the well (of resources) is high while the potential of the sink (where scums accumulate) is low. In a thermodynamic system, the potentials thus introduced are intensive variables, corresponding, e.g., to specific Gibbs chemical potentials. Consequently we define respectively $\mu _H$ and $\mu _L$ for the potential of the well and the sink.

High potentials account for the capacity of raw inputs to yield end use goods and services, while the same resources lower the potential difference when contributing to the waste flux. We shall therefore relate potentials respectively to the available quantity of resources and to the secondary residual (the “waste”) stock. For a conversion engine, input and output are usually thought of as energies, and efficiency can be defined as the output work to input energy ratio. A representation of such a heat conversion operation is given in Fig. 1a. We can show, using the finite time thermodynamics framework, see^68^, that power and efficiency of production are functions of intensity, with heat power as typical resource and sink, and mechanical power as output. It is therefore possible to plot one against the other, as shown in Fig. 1a. At low intensity, efficiency and power increase proportionally. Then the curve flexes and the maximum efficiency point is reached. If intensity increases further, then the maximum production point is reached, at the Cexpense of a significant decrease in efficiency. Beyond this point, efficiency and production power collapse. The conversion machine is then approaching exhaustion condition, in the metabolic sense of the term. Each sheet of the physical world composing our model is supposed to work between the points of maximum efficiency and maximum power. However, the economy-based control proposed in this model makes it possible to force the production intensity at will.

### Useful work and dissipation

A substantial literature has shown that many phenomena (including living ecosystems) are dissipative structures, that is, complex out-of-equilibrium systems that can only be maintained with a flow of E &M; see^48^. Non-living dissipative structures exist likewise: tornados, a pot filled with water on the fire, etc. At the scale of the complete biosphere, this dissipative structure works as a thermodynamic thermal engine which follows cycles of transformation using the Sun as a primary hot source and the night sky as an ultimate cold source; see, e.g.^69^, and the references therein. All the living systems of the biosphere are located in the trophic chain, from photosynthetic vegetal receiving their energy from the solar flow, to herbivores that feed from solar energy stored into the vegetal matter as chemical energy, to carnivores that benefit from a very concentrated chemical energy stored in meat. From there, one can provide some accounting of matter relying on energy units.

The union of two dissipative structures being still a dissipative structure, a living human society can be viewed as well as an out-of-equilibrum dissipative structure: it receives E &M in the form of biomass, raw matter, fossil energy, geothermal energy wind, and sun light and converts it into work and trash. Even more, it *must* constantly produce some work in order to maintain, or increase, the complexity of its physical structure. Following^70^ and^15^, one metric for the type of work performed by human societies is provided by *useful work*. The latter focuses on primary energy converters, grouped into the following four categories:

1. Muscle work
2. Electricity (electrochemical energy)
3. Mechanical drive
4. Heat (low, mid, and high temperatures)

Useful work is therefore a generalization of mechanical work, not to be confused with gdp (or national income) measures. It includes both manufactured goods and services and underground economic and non monetary transactions. Loosely speaking, one could argue that useful work captures the physical counterpart of what the economic concept of gdp stands for. It must be stressed, however, that even “real gdp” is a monetary concept which depends upon a given price system, while useful work is a purely physical quantity. In the sequel, *G* stands for the transformed fraction of a given flow of E &M that contributes to useful work. The flow, *G*, is directly related to the incident and outgoing flows, named $F_{H}$ and $F_{L}$, and their associated high and low potentials, $\mu _H$ and $\mu _{L}$.

The incoming and outgoing flows, $F_{H}$ and $F_{L}$, and their associated potentials, $\mu _H$ and $\mu _L$, define the incoming entropy flux $\overset{\varvec{.}}{S}_H=\frac{F_{H}}{\mu _H}$, as well as the outgoing one, $\overset{\varvec{.}}{S}_L=\frac{F_{L}}{\mu _L}$. Under ideal reversible conditions, also called Carnot conditions, we get $\overset{\varvec{.}}{S}_H=\overset{\varvec{.}}{S}_L$: efficiency reaches its maximum $\eta = \frac{F_{H}-F_{L}}{F_{H}}=1-\frac{\mu _L}{\mu _H}$, obviously lower than unity (Novikov-Curzon-Ahlborn-type (NCA) endoreversible machines are connected to the reservoirs via coupling resistors. These are then in mixed coupling condition. In this work, we have not specified a coupling resistor. However, the value of the potentials depends on parameters that can be chosen by the user. Here, for simplicity, we restricted our purview to the quantity available in the reservoirs (i.e. the reservoirs level), see Eqs. (8) and (9). So, although the potential difference to which the conversion zone is subjected is not modulated by coupling resistors, it varies and induce and intrinsic feedback by variations in the quantities, and extrinsic feedback by the possible presence of recycling, as would be the case in a NCA configuration. Interestingly, production-potential coupling could lead to spontaneous oscillations^71^). Notice that the maximal efficiency of a conversion process never coincides with its maximal instantaneous production. For the most efficient systems—efficiency being measured by the ratio between production and resources—the intensities required for maximal efficiency and maximal production are very different. Paradoxically, only poorly efficient systems exhibit some proximity between these two maxima. In other words, it is very difficult for a dissipative structure to run at its maximal efficiency and then switch to its maximal production. This observation is well known and is theoretically described as the trade-off between adaptation and adaptability, as reported by^33^; see Fig. 1a.

## Conversion engine in an economic context

The global features of an economy are depicted in Fig. 1b). The stock, $X_H$, of primary (raw) resources (regenerating or non-renewable E &M, such as coal, oil, gas, nuclear, biomass, soil, minerals ...) is represented at the top of the diagram. Its availability as an input is accounted for by the high potential, $\mu _H$. A flux, $F_{H}$, of raw resources enters the economic metabolism, represented by the ellipse. At the bottom, the used resource, $X_W$, denotes the secondary residuals or “wastes.” Its potential accounts for its output sink capacity. Primary resources (E &M) as well as flows and residuals throughout the economy, are measured in physical units (such as joules and g) and not in monetary terms.

### Production sphere

The economy operates as a conversion engine, producing a flow of useful work, *G*, and rejecting a flux, $F_{L}$, of residuals (emissions or debris) into the sink (atmosphere, ocean, land, forest...), represented by a stock, $X_W$. The flow *G* refers to the E &M transformed by manufacturing goods and services as well as infrastructures. A flux of degradation, $R\,J^2$, accounts for the internal friction of the conversion engine. As a friction term, *R* measures the fraction of E &M lost during the conversion. It is therefore directly linked to the physical production capital (roads, machines, housings, utilities...). If the latter benefits from technological or organizational innovations, and is regularly subject to investment, then *R* can keep a low value. As we shall see below, capital undergoes a natural erosion over time which, in the absence of investment, gives rise to the climb of *R*, hence, of the proportion of energy and materials lost.

The throughput, *G*, can either directly satisfy aggregate demand or, if production exceeds current demand for resources, $G_D$, fill the stock, $X_S$, of inventories and installed capacities (named buffer stock. For clarity, we have disentangled capital production from the buffer stock.) fluxing its energy via $G_S$. While a fraction of *G* can be piled in a stock of inventories and infrastructures, there is, as we shall see, no ready-to-use stock of final goods and services: following standard accounting practices, consumption deals only with non-durable commodities and services while durable goods are accounted for as investment (in $X_S$). Of course, whenever needed aggregate demand, $G_D$, draws both on the buffer stock, $X_S$, and on the current production *G*. In both cases, the incoming flux of E &M is converted before being dispersed into $X_W$.

The global intensity of economic activity is ${\mathcal J}\ge 0$ but, for each sheet, a specific intensity $J=m\, {\mathcal J}$ characterizes the rate at which material resources are converted. For $m > 1$, the subsector under scrutiny works at a larger intensity than the global economy, while for $0\le m < 1$, the subsector is metabolizing E &M less intensely. Whenever *m* shrinks to zero, the economic “body” shrivels up and “dies”. As part of a physical metabolism, no production sector can react instantly to an upheaval of exertion when internal demand for power expenditures climbs substantially. Thus, each sheet is assigned a characteristic response time, $\tau >0$, that acts as a low-pass filter against too-fast changes in economic intensity. The operating intensity of a sheet is therefore governed by a differential equation whose solution, *J*, is the sheet’s response to the intensity, $J^D$, requested by the overall economic sphere (see Eq. (3) infra). This aspect of our model is based on the work of Lars^72^ on the locally linear thermodynamic response.

Note, in particular, the analogy with thermal machines, in which incident energy flows are proportional to the entropy potentials and flows. In the present case, $F_{H}$ is proportional to $\mu _H$ and to the intensity, *J*, which is a proxy for entropy transportation^73^. The equations undergirding the functioning of a sheet are (each variable being indexed by time):

1 $$\begin{aligned} X_T=\,\, & {} {{X_H+X_W+ X_S }} \end{aligned}$$

2 $$\begin{aligned} J=\,\, & {} q \, {\mathcal J}, \end{aligned}$$

3 $$\begin{aligned} J^D=\,\, & {} \tau \, \overset{\varvec{.}}{J}+ J, \end{aligned}$$

4 $$\begin{aligned} F_{H}=\,\, & {} \mu _H \, J, \end{aligned}$$

5 $$\begin{aligned} F_{L}=\,\, & {} \mu _L \, J + {R} \, J^2, \end{aligned}$$

6 $$\begin{aligned} G=\,\, & {} F_{H} - F_{L} \end{aligned}$$

with *q*, $\mu _H$, $\mu _L$, $R, \tau \ge 0$. Equation (1) expresses that the total amount, $X_T$, of a given resource is constant across time: only its distribution between its source, $X_H$, its wastes, $X_W$, and the buffer stock , $X_S$, of unsold products and installed capacities varies. As energy is the universal measure of matter transformations, resources are dimensioned as energy.

According to (4), if $\mu _H$ is high, access to resources is easy and the flow, $F_{H}$, will be large whenever resources are harnessed intensely. The potential, $\mu _H$, however, exerts no influence the quantity of rubbish. On the other hand, if intensity, *J*, is high, the amount of produced wastes will also be sizeable. This is expressed by Eq. (5) which further states that the amount of pollution will increase more than linearly with respect to *J*. Hence, the quadratic element in Eq. (5) which is typical of viscous frictions and of Ohm’s law^67^. The last equation is again a consequence of mass and energy conservation: the flux of useful work is the difference between input and output flows.

Obviously, intensity is an essential variable since it defines the rate at which resources are used. If it is too high, the reservoir may not have enough time to regenerate, and the whole production system may abort in the medium run (an example of such a behavior is given in “Recycling impact” section).

Equation (5) implies that production is irreversible as soon as $R>0$. As remarked by^46^, this means that the metabolizing process exhibits decreasing (biophysical) returns to scale. By contrast, all the economic literature devoted to constant returns-to-scale production implicitly postulates that $R=0$. A nonzero friction term also contrasts, e.g., with^74,75^, where human “capital” is viewed as an ultimately reversibly produced good. We insist, on the contrary, that the human body is likewise a dissipative structure which exhibits no reversible metabolizing protocols, and will never be able to do so. As an example of a non-reversible technique, current photovoltaic cells currently achieve $\sim$20% efficiency, the absolute (quantum) limit for any conceivable solar device being $\sim$70%. Overall, today, about 2/3 of all energy utilized by humans is wasted and immediately dissipated into the environment^76^. One consequence is that, beyond greenhouse-gas-related climate change, waste heat also contributes to global warming. Although the total anthropogenic heat pollution flux is currently negligible ($\sim$23 TW to be compared with $\sim$120,000 TW of daily solar insolation), statistically significant continental-scale surface warming of +0.4–0.9 °C is forecast by 2100 due to dissipated energy from urban heat islands^77,78^. Furthermore, waste heat (governed by the 2nd law) is not the unique cause for pollution: according to the 1st law, “*all* energy used by the anthroposphere (efficiently or not) [will] eventually dissipate into the air at some temperature”^76^. Here, this will be accounted for by $R>0$ and capital depreciation, $\delta >0$ (to be defined shortly).

In this context, observe that (6) holds even for an isothermal economic structure far from any thermodynamic equilibrium and even for fast protocols, as is proven by Jarzynski’s identity^79,80^, provided *G* be understood as an average taken over a non-equilibrium ensemble of individual trajectories (of finite duration) of microscopic states. Throughout the paper, we keep the notations of classical thermodynamics, bearing in mind, however, that “work” and other macro-quantities are always understood as averaged magnitudes.

Notice that (averaged) work, *G*, is a parabolic function of *J* presenting a maximum at $J^\text {max}=(\mu _H - \mu _L)/2 \, R$ and zero production at $\overline{J}_p=2 \, J^\text {max}$. That $\overline{J}_p$ be finite means that maximizing gdp growth (as a monetary counterpart to maximizing *J*) does *not* boil down to maximizing useful work.

Whenever demand exceeds supply and the buffer stock is empty, aggregate demand is rationed, so that the fulfilled part of demand, $G_D^S$, reads:

7 $$\begin{aligned} G_D^\text {S}:=&{\left\{ \begin{array}{ll} G_D &{} {\left\{ \begin{array}{ll} \text { if } G-G_D>0 \\ \text { or } X_S>0 \end{array}\right. }\\ G &{} \text { otherwise. } \end{array}\right. } \end{aligned}$$

How should we define potentials? The higher the reservoir, $X_H$, of resources, the higher the potential $\mu _H$; the same holds for $X_W + X_S$ and $\mu _L$. For instance, one can adopt,

8 $$\begin{aligned} \mu _H:=\,\, & {} \tanh \left( \alpha \, \frac{X_H}{X_T}\right) \end{aligned}$$

9 $$\begin{aligned} \mu _L:=\,\, & {} \tanh \left( \alpha \, \frac{{{X_W + X_S}}}{X_T}\right) \end{aligned}$$

with $\alpha >0$. Accumulation of capital and/or wastes results in an increase in $\mu _L$, which therefore measures the filling of Earth’s atmosphere-ocean system with materials that are not ready for human use.

Initial conditions and parameters are chosen such that $\mu _H > \mu _L$ holds true throughout the time evolution of stocks. (Note that $\alpha =1$ works for all our computations.) The choice of the hyperbolic tangent stems from the fact that $\mu _*$ ($*= H, L$) must, first, locally increase linearly with $X_*$, and then saturate from above. Our contention is that sigmoidal, S-shaped curves seem ubiquitous in biology and geophysics—think of microbes replicating unsustainably in a Petri dish or of the human population plateauing later this century^81^—and might often be the symptom of a similar shape in resource potentials. It is therefore clear that these two potentials are not directly intended to account for the thermochemistry of E &M, but should rather be viewed a probe of the state of resources and wastes whose dynamics enables to estimate the evolution of the cost of harnessing stuff and recycling rubbish. Table 2, based on data from^82^, provides an estimate of these potentials for a number of critical minerals in Europe. It confirms *inter alia* that, despite its crustal abundance, copper is a critical mineral (see, e.g.^83^) since its potentials’ gap is relatively low compared to that of many other minerals.

**Table 2:** Estimated values of the potentials for selected commodities, assuming $\alpha =10^{3}$, and based on the work^84^ of Calvo et al.

Mineral	$\mu _H$	$\mu _L$
Aluminium (gibbsite)	0.96	0.88
Cadmium	0.72	$1.1\cdot 10^{-4}$
Cobalt (linnaeite)	$2.7\cdot 10^{-3}$	$5.1\cdot 10^{-6}$
Copper	0.99	0.76
Gallium (in bauxite)	0.99	0.02
Germanium (in zinc)	0.44	$1.4\cdot 10^{-3}$
Gold	0.52	$1.3\cdot 10^{-6}$
Iron ore	0.87	0.75
Lithium	0.44	0.37
Manganese (pyrolusite)	0.1	0.05
Nickel (sulfides) pentlandite	0.94	0.06
Palladium	0.46	$3.9\cdot 10^{-7}$
Phosphate (rock) apatite	0.99	0.38
Platinum	0.46	$3.9\cdot 10^{-7}$
Potassium	$6.7\cdot 10^{-3}$	$2\cdot 10^{-3}$
Silver (argentite)	0.99	$1.2\cdot 10^{-5}$
Sodium (halite)	0.99	0.53
Uranium	0.44	$1.5\cdot 10^{-3}$

The high (resp. low) potential is estimated as $\mu _{H}:=\tanh (x_c/x_m)$ (resp. $\mu _{L}:=\tanh (x_c)$, with $x_m:=r/m$ average density of reserves with respect to world mines (in g/g), and $x_c:=r/c$, the crustal concentration of reserves (in g/g). This amounts to $X_H/X_T=m/c$ and $X_W/X_T=r/c$, with $X_S=0$, for simplicity.

Note that our sheet approach excludes direct substitutability between used resources. Therefore, a resource is used up to depletion, while being piled up as a durable facility or filling the secondary residuals’ sink. Consequently, n economic dissipative structure can be constrained either by resource exhaustion or by too much trash and infrastructures. This is consistent with thermodynamics, as previously stated in “Thermodynamics conversion and boundary conditions” section (the conjunction of these two phenomena was also at the heart of^85^, although this report did not frame its set-up in thermodynamic terms). In order for the economic metabolism to remain E &M-intensive and yet wasteful in the long run, one needs to add a recycling process, efficient enough to regenerate resources and drain the waste reservoir. This will be represented by the two right-hand-side blocks of Fig. 1b and described in “Recycling” section.

### Potentials, entropy, and exergy

This subsection clarifies a few points concerning the thermodynamic measurement of resource quality. They are best illustrated by Carnot’s yield, which stipulates that efficiency of a heat engine is necessarily lower than Carnot’s factor $(1-T_c/T_h)$, where $T_c$ and $T_h$ are the cold and hot tank temperatures respectively. The central issue, which alone sums up the singularity of thermodynamics, is that of the gauge. Indeed, in most physical disciplines, the choice of a gauge, i.e., the zero of the measuring scale, is arbitrary: think of the ground potential in electrokinetics or the zero altitude at the sea level in mechanics, not even mentioning the various gauges that shape the four fundamental interactions. Things are radically different in thermodynamics, where a gauge is rigidly defined by the temperature scale called thermodynamic temperature. If not, it would be possible to assign any arbitrary value to $T_c$. Choosing $T_c=0$, Carnot’s efficiency would be always equal to unity: thermodynamics would lose its *raison d’être*. The 2d principle and the very notion of entropy thus boil down to the existence of an indisputable fundamental gauge. The concept of exergy then immediately follows from Carnot’s factor: it is only a “dual” representation of the 2d principle, in which the fraction of energy really usable in the form of work—also called free energy—is the total available energy multiplied by Carnot’s factor. However, and for practical reasons, the exergy approach often relies on a chosen reference temperature, which amounts to defining an arbitrary gauge. As a consequence, in our view, the notion of exergy is only a loose translation of the 2d principle, whereas the entropy concept encapsulates the full power of the 2d law. In other words, the more dispersed the initial material (high entropy), the larger the exergy fraction (low entropy) of energy input is needed to power its transformation into a finished product (low entropy). This is all the more pronounced when the initial material is in divided form. As a illustration, the metallurgy of mineral resources requires an essentially thermal input, which has a rather low exergy fraction, limited by the Carnot factor. On the other hand, photosynthesis, which proceeds with extremely divided matter (very high entropy), is dependent on solar photons, whose exergy fraction is very high (very low entropy).

If the question of the energy gauge is rather simple because it is based on the very definition of the thermodynamic temperature, the same cannot be said for material resources. As already mentioned, the 2d principle also applies: like energy, matter is subject to dispersion with the irreversible march of time. The measure of an element of matter within its environment is defined by its chemical potential. This, however, does not deliver an absolute gauge for matter, i.e., an absolute zero of chemical potentials. An elementary reasoning supported by an equally simple thermodynamic calculation shows that the energy cost for the concentration of a diluted resource diverges as a logarithm function with the dilution rate. Is there a gauge that would characterize ultimate dilution, i.e., the disappearance of matter? Because of the 1st law, no absolute gauge can qualify matter in the strong sense of the 2d law, such as the one that exists for temperature. Whereas there is indeed a scale of absolute temperature, there is no absolute scale of chemical potentials. If, now, a loose version of the 2d law is accepted, material qualification can be considered in terms of energy efficiency. This approach has been developed in the last 20 years by Valero, and before him by Ayres, who first introduced the concept of exergy for matter in ecological economics. At the scale of planet Earth, a gauge is usually given by the ultimate concentrations that can be found in the Earth’s crust, oceans, or atmosphere. A standard thermodynamic calculation then allows to evaluate the energy needed for a diluted chemical element to recover its initial ore density. Such a strategy may seem quite realistic in that it is loosely in perfect agreement with the laws of thermodynamics.

However, three criticisms can be formulated. The first one, already alluded to, concerns the weak form of the 2d law epitomized in the exergy-oriented approach: as soon as the gauge becomes an adjustable parameter, the exact relation to thermodynamics weakens to the point that whether treating matter in terms of energy is more relevant than another viewpoint becomes questionable. The second critique comes from the numerical estimates that emerge from these calculations and their economic validity. Indeed, the energy cost of recycling a diluted resource becomes dependent on the choice of gauge, which does not allow its accuracy to exceed an order of magnitude. Surely, the dependence is logarithmic so that the sensitivity to the gauge choice is limited for a wide range of concentrations, except for concentrations close to the minimal value—...set by the gauge itself. Third, while the energy cost of concentrating a resource can be estimated, yet nothing is said about the quality of energy required. In certain favorable situations, simple fusion-aggregation may suffice, which boils down to using only heat. In other cases—e.g., osmotic separation processes—, some input of work will be indispensable. The sustainability of living organisms results from the supply of very high quality energy in the form of solar photons whose electromagnetic energy is converted by plants into chemical energy only at a rate of $\sim$0.1%: a simpler supply of heat in equal quantities would not permit to close the cycle of living organisms via photosynthesis. As everywhere in thermodynamics, it is through a high quality energy input that cycling processes, which are counter-entropic by definition, are carried out. Adding this quality dimension to the discussion requires reasoning in terms of entropy—not just exergy—at the cost, however, of lacking an absolute gauge for matter.

This is why we refrained from choosing a thermochemical formulation of resource potentials in  (8) and (9). Although they are necessarily somewhat tainted by arbitrariness, such normalized potentials enable an assessment of (*i*) resource availability, (*ii*) the not-readily-available trash, (*iii*) hence, the criticality of a resource, and (*iv*) a quantitative criterion for the need to recycle. The difference in potentials, $\Delta \mu {{:=}}\mu _H-\mu _L$, acts indeed as a driving force for economic conversion. This gradient is the primary cause that makes it possible to transform E &M, whether in manufacturing production, in delivering services, or in the production of living organisms. In living organisms, this is nurtured by the flow of solar energy, which, being of high quality, makes it possible to maintain a difference in chemical potential throughout the trophic chain. Were this flux to deteriorate (quantitatively or qualitatively), the matter located in the biosphere, especially living dissipative structures, would inexorably end up dispersing. This is similar to what is encountered in photovoltaic conversion, see Alicki^86^.

Observe as well that, again, it is the high quality of solar power which fuels natural recycling—an incomplete process when dealing with matter^87^. This is an invariant feature of thermodynamics: lowering the entropy of a system requires a very high quality energy expenditure. This applies beyond the case of natural recycling: manufacturing production can only become cyclical provided it enjoys an influx of high quality energy, ensuring forced recycling by guaranteeing that the potential difference, $\Delta \mu$, will not vanish. Indeed, the eventual pinching over time of $\Delta \mu$ is a major determinant of a crisis in (re)production. Of course, and for a limited time period, fossil fuels may contribute to widen this $\Delta \mu$ difference via a forced recycling that takes advantage of solar energy concentrated over millions of years—this is but what the industrial revolutions have mainly been about. But, as we shall see shortly, the drawbacks are important.

In each sheet, the quantities $X_*$ and the qualities $\mu _*$ provide a full-blown description of resource consumption and recycling, exergy being given by $E_x:=\frac{\Delta \mu }{\mu _H}$. Besides, according to Onsager^72^, in a close to equilibrium thermodynamic derivation, the production of entropy is given by the product of the force, $1/\mu _*$, by the flux, $F_*$. The balance of the incoming and outgoing entropy flows during production in a sheet is therefore simply given by

10 $$\begin{aligned} \overset{\varvec{.}}{S}=\,\, & {} \frac{F_{L}}{\mu _L}-\frac{F_{H}}{\mu _H}= \frac{R \, J^2}{\mu _L} \end{aligned}$$

For a reversible production process, one would get $\overset{\varvec{.}}{S}=0$, hence $\frac{F_H}{\mu _H}=\frac{F_L}{\mu _L}$, or equivalently, $R = 0$.

Minimizing the *R* impedance can be understood as a means, for an energy conversion system, to maximize the convective transport of matter. As recalled by^33^, this convective transportation is at the very origin of the conversion of energy. When the biosphere is understood as a meta-system of energy conversion, it is sometimes suggested that the operating point must maximize power produced, erected as a principle of maximum MPP power^32^. In addition, an evolutionary tendency for complex systems is empirically observed to move towards an intensification of the free energy density (normalized to bodily mass) that runs through them^88^. Notwithstanding many exceptions to any such proposed biological “law”, such a widescale trend agrees with free energy flow maximization being the dual form of convective flow maximization, i.e., minimization of *R*. However, to claim that this propensity corresponds to a MPP is questionable^89^. Rather than ideal parameter values, our approach addresses ranges in E &M rates below which the economic metabolism is starved and above which it self-destroys. This observation might seem rather partial because it concerns only one variable, *R*. However, each sheet has its own friction, *R*, and a concrete economy is made up of millions of such sheets. Global minimization of the entropy production at the scale of an economic system thus emerges from complex trade-offs between the various frictions occurring within this myriad of sheets.

Since there is no such thing as self-organization without high quality energy input, this requires an additional extraction of energy from the reservoir. Considering a unique sheet we get the following differential equations in addition to (1)–(6):

11 $$\begin{aligned} \overset{\varvec{.}}{E}_{H}=\,\, & {} F_{H}\; \left( 1-\frac{\mu _{L}}{\mu _{H}}\right) =\Delta \mu \, J \end{aligned}$$

12 $$\begin{aligned} \overset{\varvec{.}}{E}_{L}=\,\, & {} F_{L} \; \left( 1-\frac{\mu _{L}}{\mu _{L}}\right) =0 \end{aligned}$$

13 $$\begin{aligned} \Delta \overset{\varvec{.}}{E}_{X}=\,\, & {} \overset{\varvec{.}}{E}_{H}-\overset{\varvec{.}}{E}_{L} =\Delta \mu \, J \end{aligned}$$

14 $$\begin{aligned} \eta=\,\, & {} \frac{G}{F_{H}} \end{aligned}$$

15 $$\begin{aligned} \varepsilon=\,\, & {} \frac{G}{\overset{\varvec{.}}{E}_{H}} \end{aligned}$$

The incoming exergy flux writes $\overset{\varvec{.}}{E}_{H}$; $\eta$ measures energy efficiency, and $\varepsilon$ the exergy efficiency. Equation (12) means that rubbish no longer contain any exergy. Some expressions are of specific interest. First, the maximal useful work $G_{\text {max} } = \frac{\left( \Delta \mu \right) ^{2}}{4 \, R}$ is associated with the intensity $J^\text {max}=\frac{\Delta \mu }{2 \, R}$. Second, the entropy fluxes and the entropy production, $\overset{\varvec{.}}{S}$, are given by

16 $$\begin{aligned} \overset{\varvec{.}}{S}_{H}=\,\, & {} \frac{F_{H}}{\mu _{H}} = J \end{aligned}$$

17 $$\begin{aligned} \overset{\varvec{.}}{S}_{L}=\,\, & {} \frac{F_{L}}{\mu _{L}} = J + \frac{R \, J^{2}}{\mu _{L}} \end{aligned}$$

18 $$\begin{aligned} \overset{\varvec{.}}{S}=\,\, & {} \overset{\varvec{.}}{S}_{L}-\overset{\varvec{.}}{S}_{H} = \frac{R\, J^{2}}{\mu _{L}} \end{aligned}$$

As already said, useful work, *G*, follows a short-circuit configuration from $G(J=0)=0$ to $G(J=\frac{\Delta \mu }{R}) = 0$. According to a general thermodynamic approach taken by^33^, we can identify three characteristic intensities along this curve. Increasing *J*, we first reach the point of maximal energy efficiency. Then, the already mentioned maximal production (maximal power) point is attained. Beyond $G_\text {max}$, both production and efficiency decrease—an obviously detrimental scheme. As for exergy efficiency, $\varepsilon$, it may be rewritten

19 $$\begin{aligned} \varepsilon=\,\, & {} \frac{G}{\overset{\varvec{.}}{E}_{H}}=1-\frac{J}{2 \, J^\text {max}}. \end{aligned}$$

The variable $\varepsilon$ is, of course, neither constant across time nor identical across countries. For large industrial economies, its mean, however, seems to have been orbiting around $\varepsilon \sim 0.19$ in the early 2000s’; see Ayres^15^. According to (19) we get the value of the average intensity of the production $J=1.62\times J^{\text {max}}$. This suggests hat the system was then working well beyond its maximal production zone, in a detrimental region where both production and efficiency were far from being optimal.

### An estimation of *G* for the world economy in 2010

According to^15^, the ratios, $$\rho$$, between useful work and real gdp for the US, UK, and Japan were remarkably close to each other in 2000, around $$\sim$$1.6 MJ/US$. Despite the idiosyncratic stories of each of these countries, all these country-specific ratios where declining since their peak around 1970 at a pace that should have led them close to 1.5 MJ/US$ 10 years later, provided their dynamics remained roughly similar. In 2010, the world gdp was US 65.96 trillion. Extrapolating $$\rho \sim$$ 1.5 MJ/US$ for the entire world, an educated guess leads to a useful work $$\sim$$ 98.33 eJ/a, hence 2.35 Gtoe in 2010. Now, we need to take account of the fact that, by construction, the gdp metric neglects the informal sector—which obviously also relies on energy and matter. The informal sector (including the underground economy, illicit activities like prostitution and the sale of drugs and weapons) is estimated to represent 8–10% of the gdp in the US, and more than 70% in Ivory Coast. More generally, informal transactions are often estimated to account for one-third of the total economy in Southern countries and slightly more than 10% of the total economy in advanced countries. We therefore get a back-of-the-envelope educated guess of *G* between 108.17 and 127.83 eJ/a for the world economy in 2010.

### Recycling

Once the resource is used and partly transformed into secondary residuals, it can be recycled either (*i*) through some forced recycling that runs like production (the central zone of Fig. 1b, with the engine $R_R\, J_{R}$); or (*ii*) through natural recycling (depicted by the arrow with flux $F_{NR}$ at the right hand side of Fig. 1b). Natural recycling mainly concerns primary biomass but it also includes reproduction for animals, plants, bacteria... Forced recycling mainly deals with debris that are unable to recycle endogenously on their own and therefore require human technical assistance to be re-transformed into usable stuff. For simplicity, we consider that recycled matter possesses the same qualities as that of the pristine resource, see Ayres^90^—even though this is obviously not true for metals, for instance.

For the forced recycling zone, the system of balance equations is similar to system (2)–(6):

20 $$\begin{aligned} J_{R}=\,\, & {} n_R \, J \end{aligned}$$

21 $$\begin{aligned} F_{HR}=\,\, & {} \mu _H \, {J_{R}} \end{aligned}$$

22 $$\begin{aligned} F_{LR}=\,\, & {} \mu _L \, J_{R} - R_{R} \, {J_{R}}^2 \end{aligned}$$

23 $$\begin{aligned} F_{RIn}=\,\, & {} F_{HR} - F_{LR} \end{aligned}$$

In order to make the recycling engine work, a flow of primary resource, $F_{RIn}$, is required. The input flow of residuals into the recycling engine is denoted $F_{LR}$. A flux of recycled resource, $F_{HR}$, is then injected back to the high-potential reservoir. Intensity, $J_{R}$, in Eq. (20) and friction coefficient, $R_{R}$, respectively parallel the *J* and *R* parameters of the production zone. All the flows under scrutiny are assumed to be positive, and we define $J_{R}^{\text {max}}$—see (24)—as the maximal possible intensity of the recycling metabolism which enables to get a maximal $F_{LR}$ in (22):

24 $$\begin{aligned} J_{R}^{\text {max}}:=\,\, & {} \frac{\mu _L}{2 \, R_{R}} \end{aligned}$$

The natural recycling flow, $F_{NR}$, features the renewal of a resource by natural channels—e.g., reproduction in the case of biomass. In the literature, the natural rate of growth is governed by the very classical Verhulst logistic equation, $F_{NR} = r \, X_H \, (1 - T_H)$, with $\displaystyle {T_H= \frac{X_H}{X_T}}$ and *r* being the regeneration rate. Several ecological studies^91^ point out that, for many species, the fertility rate, *r*, decreases when the population is excessively reduced. This phenomenon is known as the Allee effect^92:^

25 $$\begin{aligned} F_{NR}=\,\, & {} r \; X_H \; \left( 1 - T_H \right) \; \left( \frac{T_H}{s} -1 \right) \end{aligned}$$

In Eq.  (25), the carrying capacity is the initial and maximum quantity of initial resource, $X_T$, and $s\in [0,1]$ is the fraction of this carrying capacity below which *r* declines. In the next subsection, we shall now write the governing equations for the stocks’ evolution. An inflow of resource in a stock is positive, while an outflow is negative.

## Resource case studies

**Table 3:** Parameters of resource case studies.

Symbol	Description	Value
$X_T$	Total quantity of resource	1000.0
*r*	Regeneration rate	0.025
*s*	Threshold percentage	0.2
*R*	Production friction	0.001
$R_{R}$	Recycling friction	0.001
$G_D$	Constant demand	30

**Table 4:** Intensity for each of the three resource case studies scenarios.

Case/color	Name	Equation
1/blue	Maximum intensity	$J^\text {max}= \frac{\Delta \mu }{2 \, R }$
2/green	Optimum intensity	Lower positive root of (29)
3/red	Part of optimum intensity	20% of lower positive root of (29)

### Stock balance equations

The balance differential equations for the high and low stock variables, $X_H$ and $X_L=X_W+X_S$, are:

26 $$\begin{aligned} \overset{\varvec{.}}{X}_H=\,\, & {} F_{NR} - F_{H} + F_{HR} - F_{RIn} \end{aligned}$$

27 $$\begin{aligned} \overset{\varvec{.}}{X}_W=\,\, & {} - F_{NR} + F_{L} - F_{LR} + G_{D} \end{aligned}$$

28 $$\begin{aligned} \overset{\varvec{.}}{X}_S=\,\, & {} G_S = G - G_D \end{aligned}$$

Variations of the reservoir, $X_H$, are due to the inlet of recycled resources ($F_{NR}$ and $F_{HR}$) and the outlet of resources used for production, $F_{H}$, or recycling, $F_{RIn}$. The sink, $X_W$, is filled by the consumption of final goods/services (with flux $F_{L}$). Natural and forced recycling allow to partially regenerate the reservoir, with fluxes $F_{NR}$ and $F_{LR}$ stemming from the sink. Variations of the buffer, $X_S$, are due to changes either in inventories or in installed capital. For simplicity, we assume, here, that $X_S$ does not produce waste. This restriction will be dropped when making the economic sphere explicit.

Put together, Eqs. (7) and (27) are valid as long as the buffer stock remains positive, so that $G_D$ is never rationed. This is always the case in the numerical simulations to follow, and is a standard short-cut which saves us from considering rationing schemes^93,94^. Doing otherwise would lead us to define the (possibly rationed) “satisfied demand,” $G_{D}^{\text {satisfied}} := \min \{F_{H}, G_D\}$, which would introduce non-differentiable boundary issues in the main vector field under scrutiny. For the sake of simplicity, we avoid these technicalities.

### Sheets and demand function

Obviously, the real world is more complex than the single sheet proferred above. A more realistic model has to take into account many kinds of resources and their interplay. We propose to represent these complex interactions through a central kernel where a *demand function for resources* addresses to each sheet a request for E &M, which may be only partially satisfied. This demand function will coincide with a production function (in the conventional sense of economics) only whenever resources are infinite. Figure 1c presents an example of a 4-sector economy (two material resources M1 and M2 and two energy resources E1 and E2) interconnected through a central kernel.

In this section, we substantiate our model by several case studies, focusing on a single physical sheet only, for simplicity. At $t=0$, the entire amount of resource is available and ready to be converted by the economic engine, while the buffer and the sink of secondary residuals are empty: $X_H = X_T$, $X_S=X_W=0$, $\mu _H =1.0$ (say) and $\mu _L = 0$. At $t=0$, the potential difference is maximal, as is therefore initial production, given some intensity, *J*.

Intensity is chosen such that $G=G_D$ (as we shall see infra, this amounts to assuming that there is no investment), i.e., *J* solves the quadratic equation :

29 $$\begin{aligned} R \, J^2 - \Delta \mu \, J + G_D=\,\, & {} 0 \end{aligned}$$

Results obtained using Eq. (29) are illustrated in Fig. 1d. In order to equate the demand for raw material, $G_D$, and the supply flow, *G*, two intensities are available, corresponding to the two roots of Eq. (29). Unless $(\Delta \mu )^2=4RG_D$, these roots are distinct and the highest yields more waste than the lower one. A single level of intensity, $J^\text {max}$, maximizes current production at $\displaystyle {G_\text {max}= \frac{\Delta \mu ^2}{4 \, R}}$, delivering maximal power, but exhibits less efficiency, as expected:

30 $$\begin{aligned} J^\text { max }=\,\, & {} \frac{ \Delta \mu }{ 2 \, R }. \end{aligned}$$

By contrast, a preferable intensity should satisfy the required demand with minimal waste. For our simulations, we therefore postulate a minimal aggregate rationality by adopting the lower positive root of  (29) so as to reduce wastes conditionally on the satisfaction of demand, thus staying below the maximum intensity $J^\text {max}$ of  (30). Let us call this an “optimal intensity,”

31 $$\begin{aligned} J:= \min \, \Bigl \{\text {solution of } (29)\; ; \; J^{\text {max}}\Bigr \}. \end{aligned}$$

Of course, whatever being the chosen intensity, the high potential stock decreases over time in every scenario because of dissipation and the low potential stock increases as the waste reservoir fills. The intensity of recycling and the complexity of the metabolism captured through the friction, *R*, however, play a non-trivial role as we shall now see.

### Intensity impact

**Figure 2:**
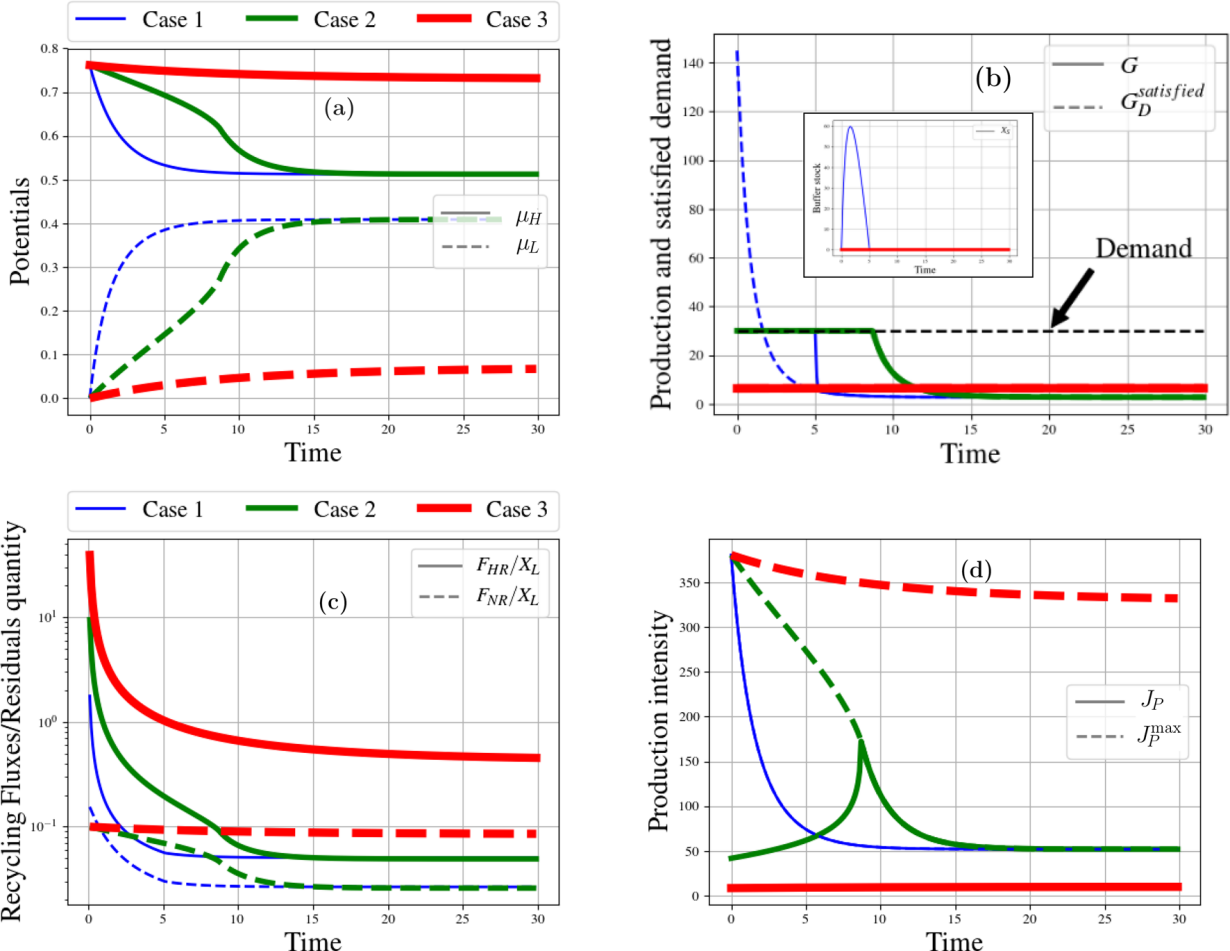
Resource case studies: intensity impact. From left to right and top to bottom. (**a**) Time evolution of high and low potentials ($\mu _H$ and $\mu _L$) for maximum (Case 1), optimum (Case 2), and weak intensity (Case 3). The dotted curve is the other positive root of case 1. (**b**) Time evolution of production of goods and buffer stock for maximum (Case 1), optimum (Case 2), and weak intensity (Case 3). (**c**) Time evolution of normalized recycling fluxes ($F_{HR}$ and $F_{NR}$) for maximum (Case 1), optimum (Case 2), and weak intensity (Case 3). (**d**) Time evolution of intensity for maximum (Case 1), optimum (Case 2), and weak intensity (Case 3).

Let us consider three cases, see Table 4: (1) the metabolism runs at the maximal intensity $J^\text {max}$, (2) at an optimal intensity, and (3) at 20% of this optimal intensity (low intensity). Furthermore we choose $q=1$. In all cases, the demand is kept constant, see Tables 3, 4.

Figure 2a plots the variations of potentials for these three scenarios.

At maximum intensity (case 1), production initially exceeds demand (Fig. 2b). Thus, the buffer stock (inset of Fig. 2a) first fills up. As long as there are enough goods in the buffer stock ($t\simeq 5$) or in the reservoir, the demand for resources is met (Fig. 2b). At optimal intensity (case 2), demand is exactly satisfied from the beginning, but then, production drops and becomes very low ($t\simeq 10$). In both scenarios 1 and 2, the gap between the potentials quickly narrows—a symptom of the fact that consumption of resources is too fast to be sustainable (Fig. 2b). The pinch-off of the potentials eventually inhibits production, which ends up in free fall as the potentials’ difference vanishes.

On the contrary, at low intensity (case 3), demand for raw materials is never fulfilled but potentials are no longer pinched. Instead, production remains nearly constant at a modest level. This is an instance of what we call “slowing down the economy” as a way to ensure (strong) sustainability. Notice that, in the long-run, it does not prevent the economy from growing but at a (very) slow pace.

The curves in Fig. 2d show the time evolution of intensities (solid line) compared to the maximum intensities (dotted line). In the first case, the two intensities are of course identical and they collapse due to the rapid pinching of the potentials. In the second case, the intensity first increases in an attempt to satisfy demand but soon reaches the ceiling, $J^\text {max}$, where it can no longer meet demand. It therefore keeps following the slope of maximum intensity, trying in vain to extract the quantity of resources requested by the economic metabolism. In the third scenario, the intensity is very low and does not increase, although it remains far from its maximal value. As for recycling, in comparison to the total quantity of waste, a larger fraction $F_{HR}/X_W$ or $F_{NR}/X_W$ is recycled when the intensity remains low (Fig. 2c). Indeed, when the metabolism runs less intensely, fewer resources are used up per time unit, and more time is left to refill the reservoir both through natural and forced recycling. At maximal and optimal intensities, by contrast, wastes are produced at a faster rate (Fig. 2b) and recycling is no longer efficient enough.

### Recycling impact

**Figure 3:**
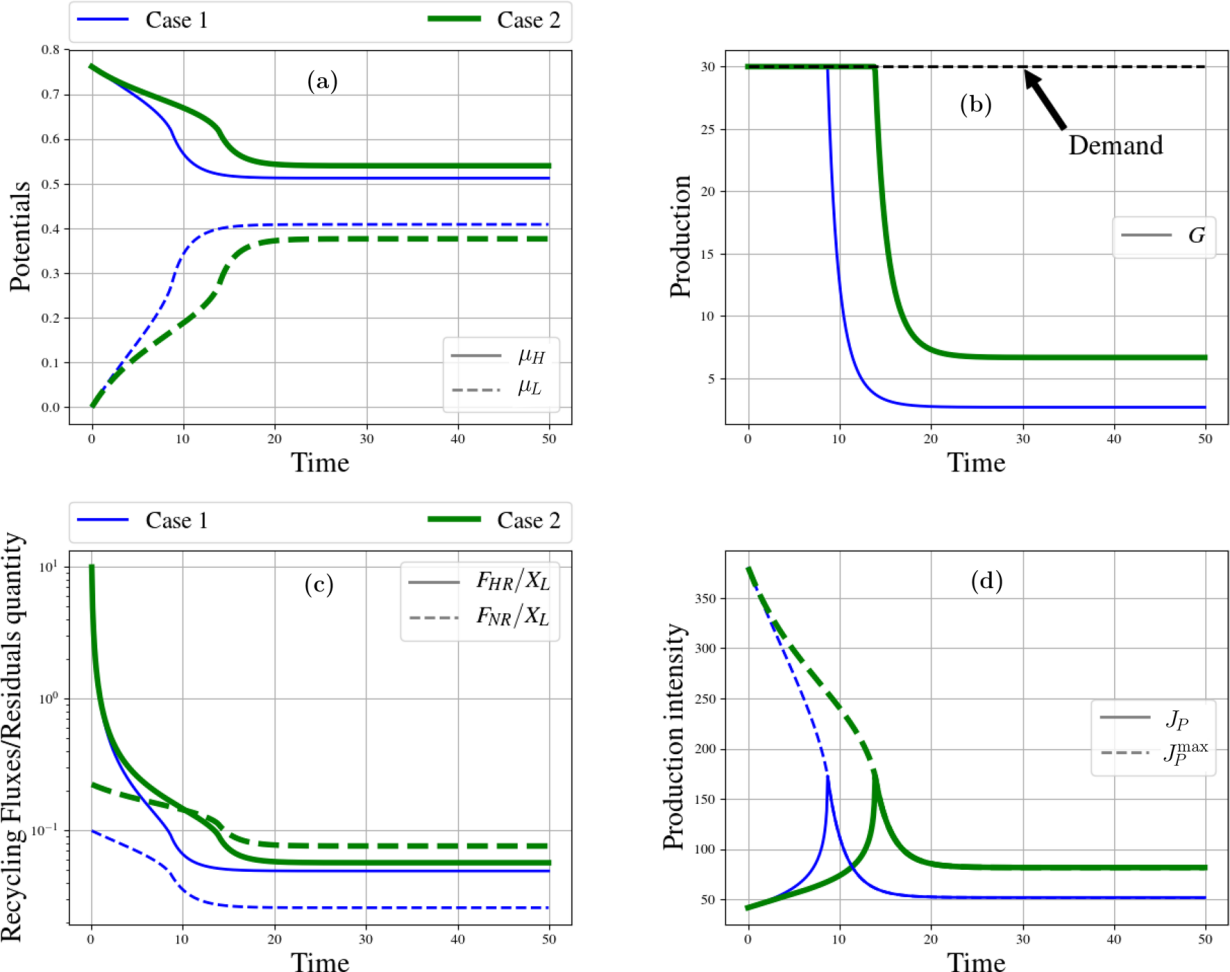
Resource case studies: recycling impact. From left to right and top to bottom. (**a**) Time evolution of high and low potentials ($\mu _H$ and $\mu _L$) for high (Case 1) and low threshold (Case 2). (**b**) Time evolution of production of goods (flux *G*) for high (Case 1) and low threshold (Case 2). (**c**) Time evolution of normalized recycling fluxes ($F_{HR}$ and $F_{NR}$) for high (Case 1) and low threshold (Case 2). (**d**) Time evolution of production intensity (flux *G*) for high (Case 1) and low threshold (Case 2).

We study two cases differing by the Allee effect threshold, *s*, that affects $F_{NR}$ (see Eq. (25)). The metabolism runs at its optimal intensity in both cases. The Allee effect threshold, *s*, is between a fraction 0.1 and 0.2 of the maximum possible E &M intake for animals^95^. The regeneration rate, *r*, lies^95^ in the range 0.01–0.05; we choose an intermediate value $r=0.025$. Scenario one deals with a high threshold at 20% of the maximum quantity, and scenario 2 with a low threshold at 10% of the maximal quantity. The result alleges that, in case 1, for $X_H <0.2 \, X_T$, thereby $T_H/s-1<0$, regeneration is harder and degrowth ends up prevailing. The higher the *s* percentage, the earlier growth rate turns negative because regeneration becomes more tedious—which explains why, in case (1), potentials are pinched earlier than in case (2) (see Fig. 3a). Indeed, natural recycling is lower in scenario 1 (see Fig. 3c). The quantities of wastes are close in both scenarios, so that forced recycling rates remain close as well.

According to Fig. 3d, intensity peaks at about 170 in both scenarios but at different times: case (2) evolves more slowly, and intensity, there, remains higher than in case (1) for a long period of time. At the beginning, as shown in Fig. 3b), in case (2), demand can be satisfied during a longer period than in case (1). This is because population regenerates at a positive rate during a longer period and enjoys a more intense flow of extracted resources. Thus, the threshold value, *s*, affects not only the short-run dynamics of a metabolism but also its asymptotic fate.

### Friction impact

**Figure 4:**
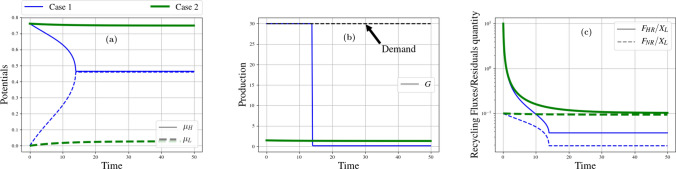
Resource case studies: Friction impact. From left to right. (**a**) Time evolution of high and low potentials ($\mu _H$ and $\mu _L$) for weak (Case 1) and strong (Case 2) friction. (**b**) Time evolution of production of goods (flux *G*) for weak (Case 1) and strong (Case 2) friction. (**c**) Time evolution of normalized recycling fluxes ($F_{HR}$ and $F_{NR}$) for weak (Case 1) and strong (Case 2) friction.

Production friction, *R*, and recycling friction, $R_{R}$, vary across time. Absent investment, their value increases over time, as a result of capital erosion. Conversely, they decrease if investments enable to improve the functioning of production/recycling facilities. Changing $R_{R}$ does not have a significant impact on our simulations since we run at $J_{R}^\text {max}$ which adapts to the variations of $R_{R}$. We hence consider only changes in *R* for which two extreme values are considered: very low: $R=4 \cdot 10^{-5}$ (case 1); and high: $R=0.1$ (case 2).

In case 1 (blue curves of Fig. 4c), the low friction enables the anabolic system to satisfy economic demand with intensity put into high. However, this seemingly satisfactory metabolizing speed is accompanied by significant pollution soiling our environmental trash bin, as can be seen in Fig. 4b. As a consequence, after a short phase of vigorous production, at “time” 13, the potentials’ differences are pinched, the garbage can saturates, the reservoir of initial resources empties, and production is suddenly on the verge of aborting. Production shortly keeps going thanks to recycling, which ensures a minute renewal of resources (Fig. 4c) but does not suffice to rebalance the metabolism against starvation. A parallel with zoology is illuminating: on average, smaller animals share a higher metabolizing intensity thanks to their “frequent eating habits, high pulse rates, robust activity levels, and relatively short life spans: they live fast and die young”^81^.

Conversely, a high friction *R* (green curves of Fig. 4a–c) makes production more tedious (see Fig. 4b) so that the gap between the two potentials only slightly shrinks as time goes (Fig. 4a). Consequently, however, both consumption of raw resources and waste delivery remain moderate. This allows for an efficient recycling (Fig. 4c). As a result, production is less flamboyant but remains sustainable in the long-run, by contrast with the low friction case. We guess from the very different physics of these two extremes that an optimal value for *R* should be located between these two extremal values—which can be interpreted as a form of impedance matching. Again, a comparison with biology helps: “Abundant deciduous trees have larger absorbing leaves that capitalize on the short, hot summers by photosynthesizing fast, yet their leaves die young compared to evergreen trees that achieve slower, steadier growth year-round” (see^81^).

### An example: the carbon cycle

Let us apply the previous framework on the specific case of carbon cycle. We rely on^96^ for the calibration of this specific version, and restrict ourselves to those variables and processes that seem indispensable in order to provide a qualitative characterization of the evolution of carbon on Earth on a time-scale of dozens to hundreds of years.

There are four sources of carbon on Earth, measured here in Gigaton of Carbon (GtC), only the first three of them being able to serve as reservoirs for human activities:

- a terrestrial (or land) carbon stock, $L\in [0,C^*]$, including soils and plants
- an atmospheric stock of carbon, $A\in [0,C^*]$
- an accessible geological carbon stock (fossil fuel reserves), $C\in [0,C^*]$
- a maritime carbon stock, $M:=C^*- A-C-L\in [0, C^*]$, which includes only the upper-part of the oceans which exchanges carbon comparatively fast with air.

Therefore, carbon totals

32 $$\begin{aligned} X_T:=C^*=A+C+L+M. \end{aligned}$$

Absent Carbon Capture and Storage (CCS), $X_H=L+C$, while (if CCS techniques were to be introduced at a significant level, $A$ would have to be treated both as a reservoir and a waste as well) $X_W=A+M$. The global mean surface air temperature $T\ge 0$ is assumed to relax instantaneously to its (thermodynamic) equilibrium value depending on *A*, using a nonlinear temperature scale so that it be simply proportional to *A*. Therefore, *T* is measured not in Kelvin but in ‘carbon-equivalent degrees’ [Ced=GtC], using an atmospheric carbon-equivalent scale: $T= x$ Ced is the equilibrium temperature of an atmosphere containing *x* GtC. Obviously, the Ced unit depends upon climate sensitivity.

The anthroposphere can extract energy from Biomass, according to the flow of carbon $B\ge 0$ [GtC/*a*], where *a*=annum. The flow of extracted biomass energy reads $E_B\ge 0$ [GJ/*a*]. In pre-industrial societies, this was the main source of energy (if one neglects windmills that spread throughout Europe from the 12th century onwards). In industrial societies, another source of energy obtains via fossil carbon extraction/sequestration: $F\in {\mathbb {R}}\; [GtC/a]$. The associated flow of fossil energy is $E_F\in {\mathbb {R}}$ [GJ/*a*]. The total energy input is $E:=E_B + E_F$.

In this carbon-dedicated specification of our model, *T* is the unique physical intensive variable, and $C^*$ is the unique conserved material quantity: Eq. (32) is nothing but (1), so that, despite its complexity, the carbon cycle can be treated within a single sheet in our paradigm. The oceans, the land, and the atmosphere all serve as a well for carbon wastes, while only land and geological resources are used as a reservoir. The main drivers of the carbon cycle are given by:

- Ocean to atmosphere diffusion $f_{\text {diff}} (A,M):=d \, (M - mA)$ [GtC/a], neglecting pressure and temperature dependency):
- Greenhouse effect on temperature: $T:=A/\Sigma$ (ignoring other GHG), where $\Sigma$ is the available Earth surace area (km$^2$);
- Land to air evapo-transpiration $f_{\text {resp}} (L,T):=(a_0+a_T \, T) \, L$ [GtC/a], neglecting other dependencies;
- Photosynthesis $f_{\text {photo}} (A,L,T):=(\ell _0 - \ell _T \, T ) \, L \, \sqrt{\frac{A}{\Sigma }}$ [GtC/a], with atmospheric carbon fertilization, ignoring nitrogen *inter alia*;
- Biomass extraction *B*(*G*, *K*, *L*) and combustion, $E_B=E_B(B)$, ignoring carbon stored in human bodies and tangible capital, and thereby assuming almost all extracted land carbon ends up in the atmosphere after a negligible time;
- Fossil fuel extraction (in industrial societies) *F*(*G*, *K*, *L*) and combustion, $F=E_F(F)$.

Estimates of the different variables used are reported in Table 5. The evolution of the carbon reservoir is given by

$$\overset{\varvec{.}}{X}_H=\overset{\varvec{.}}{L}+ \overset{\varvec{.}}{C}=(\ell _0 - \ell _T \, T) \, L \, \sqrt{\frac{A}{\Sigma }} - (a_0+a_T\,T)\,L - B - F.$$

The buffer remains constant while carbon wastes follow

**Table 5:** Overview of the model parameters and the best estimate based on real-world data^96^.

Symbol	Description	Unit	Estimate
$\Sigma$	Available earth surface area	km$^2$	1.5$\cdot 10^8$
$C^*$	Total available carbon stock	GtC	5500
$a_0$	Respiration baseline coefficient	$a^{-1}$	0.0298
$a_T$	Respiration sensitivity to temperature	km$^2 a^{-1}$ GtC$^{-1}$	3200
$\ell _0$	Photosynthesis baseline coefficient	km $a^{-1}$ GtC$^{-1/2}$	26.4
$\ell _T$	Photosynthesis sensitivity to temperature	km$^3$ $a^{-1}$ GtC$^{-3/2}$	1.1 $\cdot 10^6$
*d*	Diffusion rate	$a^{-1}$	0.01
*m*	Solubility coefficient	1	1.5
$C^*_{PI}$	Total pre-industrial carbon stock	GtC	4000
$L_0=0.72 \, C^*_{PI}$	Initial Land carbon stock	GtC	2880
$A_0=\frac{C^*_{PI} - L_0}{1+m}$	Initial pre-ind. atmospheric stock of carbon	GtC	448

Here *h* denotes the human population unit.

$$\overset{\varvec{.}}{X}_L=\overset{\varvec{.}}{A} +\overset{\varvec{.}}{M}=d \, (M-m\,A) - \overset{\varvec{.}}{L} + \overset{\varvec{.}}{M}=-\overset{\varvec{.}}{C} - \overset{\varvec{.}}{L}= -\overset{\varvec{.}}{X}_H.$$

A. *The pre-industrial case.*
  At the turn of the 19th century (where $$\overset{\varvec{.}}{C}=F=0$$), on a forested planet (for which $$L_0=0.72\,C^*_{PI}$$), Eqs. (8) and (9) lead to
  $$\mu _H=\tanh (0.72\,\alpha )> \mu _L=\tanh (0.28\,\alpha ) \; \; \forall \alpha >0.$$
  Following^97^, the world population just passed the $$10^9$$ threshold in 1800. According to^81^ “with the onset of agriculture and the use of trained animals, $$\approx -10,000$$ y, the equivalent energy available to individual *H. sapiens* (assumed here to be a 50-kg body) increased to $$\approx$$ 12,000 kcal/day [...] in turn, these would have easily doubled with the invention of advanced farming techniques and the invention of metal and pottery manufacturing a few millennia ago. (Today, the most intensive agricultural methods yield as much as 40,000 kcal per day per person.)” (p. 35) However, not every member of a farmer’s family was producing this amount of foodstuffs, so that, even though at least, 80% of the world population was working in the agricultural sector, the average daily caloric consumption cannot have exceeded 1800 kcal worldwide (see^98^ p.450). Thus, an optimistic estimate of biomass extraction in the late 18th century should be $$E_B\sim 7330$$ TJ/a. At the same pre-industrial period, the mean income per capita is estimated by^99^ as oscillating mainly between 0.5 and 1 (expressed in (2011) US$ and taking informal activities into account). Historians believe that the ratio between gdp and agricultural gross output was higher during the *Ancien Régime* than in modern times (see^100^). By how much is hard to assess, however. Ayres^101^ estimates that, around 1900, the ratio, $$\rho$$, between primary useful work and gdp was about 1MJ/(1998)US$. Since the world economy in 1800 was not concerned with secondary work (i.e., electric power producing either mechanical work or high-temperature heat), and adjusting for the US inflation between 1998 and 2011, we guesstimate $$\rho \sim 1$$ MJ/(2011)US$ worldwide, which leads to:
  $$0.5\cdot 10^3\, \text {TJ/a}\le G\le 10^3\,\text {TJ/a}.$$
  In comparison to biomass extraction, this suggests an efficiency ratio, $$G/E_B$$, somewhere between 0.18 and 0.36—which looks realistic for societies that were overwhelmingly concerned with self-subsistence. If one surmines that pre-industrialists were collectively wise enough to optimize their metabolizing intensity, *J* (and not to exceed that threshold), and adopting $$\alpha =1$$ in (8) and (9), (6) yields $$R=\frac{(\Delta \mu )^2}{4G}$$. Thus, (30) implies that worldwide anthropogenic entropy production in the late 18th century must have been located between
  $$1.85\cdot 10^3\; \text {TJ/aK}\le \overset{\varvec{.}}{S}=\frac{G}{\mu _L}\le 3.703\cdot 10^3\; \text {TJ/aK}.$$
  *Asymptotic analysis*. As shown by Nitzbon^96^, in the long run, the pre-industrial dynamics of the carbon cycle admits two locally stable steady states: a catastrophic equilibrium where the Earth turns into a desert, and a desirable one, where (neglecting external solar forcing) a human population of medieval size (approximately 200 millions) can survive for ever in a permanent Holocene on a forested planet $$(L^*\simeq 0.72 \, C^*_{\text {PI}}$$). Our model allows to guesstimate the asymptotic entropy production of this (forest) equilibrium: imploring the not-so-pessimistic working hypothesis that, in the long run, the biomass-related energy flow per capita would be identical to that of the UK in 1800 (or equivalently, the average intake in Subsaharian Africa today), i.e., 2,200 kcal/day, the biomass flow would asymptotically converge to $$F^*_B\sim 672$$ TJ/a. If the efficiency of metabolising this input into useful work stabilises atop pre-industrial levels, *G* should converge to some $$G^*\ge 242.13$$ TJ/a. Absent the Industrial Revolution, $$\rho$$ would have no reason to follow the “inverted U shape” pattern observed by^101^ and that peaked around 1970. Let’s therefore surmine that it would remain close to 1, so that the average real income per capita should asymptotically be close to (2011)US$ 1.21. On average, mankind would still live below the extreme poverty line ((2017)US$ 2.15 per day). Eventually, if our successors become wise enough to slow *J* down to its optimal value in (30), asymptotic entropy production should be close to $$\overset{\varvec{.}}{S}^*\sim 896.77\; \text {TJ/aK}.$$
B. *The industrial case* The average caloric intake of humans in 2000 (2,800 kcal/day/human), together with the fact that the world agriculture produces one third of foodstuff and textile fibers which are not consumed/used, lead to an estimated flow $$E_B\sim 45.2\cdot 10^3$$ TJ/a. Carbon forced recycling would mainly amount to CCS and methanization of farm wastes: both were certainly negligible by 2000. With a world gdp around US$ 33.835 trillions according to^102^, we get $$G\sim 50.75$$ eJ/a—which yields an estimated ratio $$G/E_B\sim 1120$$ in 2000. This, of course, is only possible because the daily energy of the Sun is no longer the main provider in industrialist societies, which instead rely on stored photosynthetic energy of fossil hydrocarbons, gravity (hydroelectric power), and terrestrial nuclear energy.
  On the other hand, it is known that mankind has emitted around 450 GtC since the onset of the Industrial Revolution and that soils have lost 133 GtC since the dawn of agriculture^103^. The impact of this change on $$\mu _H$$, however, is negligible, so that the low potential in 2000 is roughly equal to its pre-industrial value. As a consequence, $$\overset{\varvec{.}}{S}\ge 188$$ eJ/aK: a million times the order of magnitude of the entropy produced by a sustainable (preindustrial) stationary state. As will be clear below, this metabolising speed is not sustainable in the long run and likely to lead to a collapse. The central question then becomes: could we manage a more desirable fate midway between extinction and an “island” of extremely poor survivors maintaining a pre-industrial lifestyle?

## The anthroposphere

We next deploy the economic dynamics internal to our thermal conversion engine.

### Stock-flow consistency

Let us consider a simple SFC set-up with only two sectors: households and firms, hence excluding banks, the government sector, and the rest of the world. Say’s law is also assumed, in the sense that economic supply of final goods/services is postulated to be always absorbed by aggregate demand. For a more general version of the economic model with households’ debt, see^104^

The matrix of funds and flows is presented in Table 6: households’savings is given by $S^h:=-p\,C+W$, while aggregate consumption, *C* is supposed to adjust instantly so that the accounting identity, $Y=I+C$, be always satisfied, with *Y* standing for real gdp or, else, National income deflated by inflation, and *I* for investment.

**Table 6:** Economic balance sheet.

	Households	Firms		Sum
Balance sheet				
Capital		$p\,K$		$p\,K$
Transactions		Current	Capital	
Consumption	$-p\,C$	$p\,C$		0
Investment		$p\,I$	$-p\,I$	0
Memo [gdp]		$[Y=G/\rho ]$		
Wages	$w\,L$	$-w\,L$		0
Depreciation		$-\delta \,p \,K$	$+\delta \,p \,K$	0
Profits		$-\Pi$	$+\Pi$	0
Sum	0	0	0	
Flow of funds				
Change in capital			$+p(I-\delta \, K)$	$+p(I-\delta \, K)$
Change in net worth	$\overset{\varvec{.}}{X}_h=0$		$\overset{\varvec{.}}{X}_f=\overset{\varvec{.}}{p}K$	$\overset{\varvec{.}}{X}=\overset{\varvec{.}}{p}K$

### Goodwin set-up

In the present section, an elementary economic framework—namely the Goodwin model—is cast in terms of our thermodynamic sheet approach^50,51^. This economic vector field is the simplest one within a family of dynamics that have been introduced in the literature in the past 50 years, see^105^ for a survey. Let *W* stand for the wage bill of the economy, $p{{}}>0$ be the current price level, and $p{{}}\,Y{{}}$ refer to the money value of current output whereas *L* is the amount of labor performed by labor forces of size *N*. The macroeconomic module is governed by the following Lotka-Volterra system of non-linear differential equations:

33 $$\begin{aligned} \overset{\varvec{.}}{\omega }=\,\, & {} \omega \; \left[ \phi (\lambda ) - \alpha \right] \end{aligned}$$

34 $$\begin{aligned} \overset{\varvec{.}}{\lambda }=\,\, & {} {{\lambda \; \left[ \frac{1-\omega }{\nu } - \alpha - n - \delta \right] }} \end{aligned}$$

where $\nu :=K/Y= 2.89$ stands for the (constant) capital-output ratio, while $\omega :=W/(p\,Y)$ and $\lambda :=L/N$ denote respectively the wage share and the employment rate. That $\nu$ be constant is the hallmarck of labor and capital being complementary: $Y\equiv K/\nu \equiv a\,L$. One could allow for some substitutability among production factors, as in^106^, without impairing our main results: substituting labor to capital (or vice versa) would simply allow the private sector to postpone the collapse in catastrophic scenarios and to accelerate convergence towards a desirable steady state (or limit-cycle) otherwise. Finally, halfway between mere complementarity and substitutability of production factors, introducing a Putty-Clay capital structure as in^55^ would induce intermediate trajectories whose asymptotic properties are qualitatively similar to those of the two extreme cases.

**Table 7:** Initial values of macroeconomic simulations.

	Description	Initial value
$\omega$	Wage share	0.58
$\lambda$	Employment rate	0.69
*N*	Workforce	$4.55 \cdot 10^9$
*w*	Wage	11.98
*Y*	Real output level	$64.45\cdot 10^9$

**Table 8:** Parameters of the macroeconomic scenario.

Scenario	G/black	1/blue	2/green	3/red	4/cyan
$X_T$	–	$\mathbf {10^8}$	100.0	100.0	100.0
*R*	–	0.001	0.001	0.001	**0.1**
Features	Goodwin pathway	Infinite resources	Standard example	No forced recycling	High friction

The name of each scenario, line 1, appears with the same color and bold type as used in Figs. 6 and 7.

Harrod-neutral labor productivity, $a\ge 0$, grows exponentially at rate $\alpha >0$^51^:

35 $$\begin{aligned} \alpha=\,\, & {} \frac{\overset{\varvec{.}}{a}}{a} = 2.26 \cdot 10^{-2}. \end{aligned}$$

The growth rate, *n*, of labor forces, *N*, is exogenous:

36 $$\begin{aligned} n=\,\, & {} \frac{\overset{\varvec{.}}{N}}{N} = q \; \left( 1-\frac{N}{P^N}\right) \end{aligned}$$

with $P^N = 7.059 \cdot 10^9$, the upper-limit of the working population, and $q = 2.7 \cdot 10^{-2}$, its speed of growth^51^. Technological progress could be turned into an endogenous variable (as in^107^) without modifying the main qualitative conclusions of this paper. Similarly, the uncertainty around the evolution of labor productivity might be captured (as in^53^) by replacing *a* with a stochastic process. The paper just quoted suggests, however, that, while it would allow to replace our deterministic trajectories by tubular neighborhoods of possible solutions, their qualitative behavior would not be altered. The parameter, $\delta = 6.25\cdot 10^{-2}$, is the depreciation rate of capital, which obeys the standard rule of accumulation expressed in real terms:

37 $$\begin{aligned} \overset{\varvec{.}}{K}{{}}=\,\, & {} I - \delta \, K{{}}, \end{aligned}$$

where investment, *I*, is the economic counterpart of some (useful) work performed by the economic metabolism in order to harvest high-quality energy and high-entropy matter, and use the former to concentrate the latter in low-entropy infrastructures, buildings, machines, etc. Similarly, capital depreciation, $\delta \, K$, is but the economic translation of some catabolic work, $\rho \,\delta \, K$, induced by the decay of infrastructures—where $\rho :=G/Y>0$ refers to the conversion factor, introduced earlier, of economic quantities into (physical) work. Being a waste, $\rho \delta K$ contributes to filling the sink, $X_W$. We stress that our approach is flexible enough to accommodate various interpretations of capital: it can be viewed as a material stock (measured, e.g., by its mass) as in^108,109^ or, alternatively, as a flow as in^110^. When this flow is interpreted as energy, the mass-energy convertibility explains why apparently incompatible standpoints available in the literature about the meaning of “capital” are in fact reconcilable.

Consumption of final goods/services, *C*, is the economic reflection of the flow of work $\rho \, C=G_D - \rho \delta K$ (cf. (28)). Equation (27) therefore reads:

$$\overset{\varvec{.}}{X}_W = - F_{NR} + F_{L} - F_{LR} + \rho (C + \delta \, K).$$

As yet another signature of the arrow of time, capital depreciation amounts to adding a linear frictional term to the economic conversion engine. Indeed, (6) yields

38 $$\begin{aligned} K=\frac{\Delta \mu \, J \, \nu }{\rho }. \end{aligned}$$

Hence, (5) now reads:

39 $$\begin{aligned} F_{L} =\mu _L \, J + {R} \, J^2 + \frac{\delta \, \Delta \mu \, J \, \nu }{\rho }, \end{aligned}$$

whereas entropy production is given by (cf. (10)):

40 $$\begin{aligned} \overset{\varvec{.}}{S}= \frac{\rho \, R \, J^2 + \delta \, \Delta \mu \, J \, \nu }{\rho \, \mu _L}. \end{aligned}$$

Eventually, the function $\phi (\cdot )$ refers to the short-run Phillips curve. It drives the growth rate of real wages as a function of the employment rate:

41 $$\begin{aligned} \frac{\overset{\varvec{.}}{w}}{w}=\,\, & {} \; \phi (\lambda ):= \phi _0 + \phi _1 \, \lambda . \end{aligned}$$

Its parameters, $\phi _0 = -0.73$ and $\phi _1 = 1.08$, have been empirically estimated in^51^ on the world economy for the past three decades.

Following^49^ among others, prices are assumed to be given by a fixed margin on direct unitary costs. Production costs consist only of wages in this simplified model. Admittedly, further work will need to incorporate the endogenous prices of E &M. The price of goods and services, *p*, is driven by a markup $m>1$, unitary costs, i.e., the wage bill $w{{}}\,L{{}}=W{{}}$ and real output, *Y*. It reads:

42 $$\begin{aligned} p{{}}=\,\, & {} (1+m) \; \frac{W{{}} }{Y{{}}} \end{aligned}$$

In a more realistic fashion, we could also let prices relax towards the left-hand side of (42) but with no significant change in our results. Private firms’ nominal net profits, $\Pi {{}}$, depend on the difference between nominal output and the global cost of production (i.e., the wage bill):

43 $$\begin{aligned} \Pi {{}}:=\,\, & {} p{{}} \, Y{{}} - w \, L{{}}. \end{aligned}$$

Investment is entirely financed by current profits, so that,

44 $$\begin{aligned} I:=\,\, & {} Y \; (1-\omega {{}}), \end{aligned}$$

with $\Pi {{}}/(p{{}} \, Y{{}})=1 - \omega {{}}$. Consequently, the evolution of the output flow, *Y*, depends on the wage share, $\omega$, the output-capital ratio $\nu$, and capital depreciation $\delta$ (see^50,51^):

45 $$\begin{aligned} \overset{\varvec{.}}{Y}{{}}=\,\, & {} Y{{}} \; \left( \frac{1-\omega }{\nu } - \delta \right) . \end{aligned}$$

Table 7 provides the initial values of the economic system. Wages and real output level are expressed in 2010 US $.

**Figure 5:**
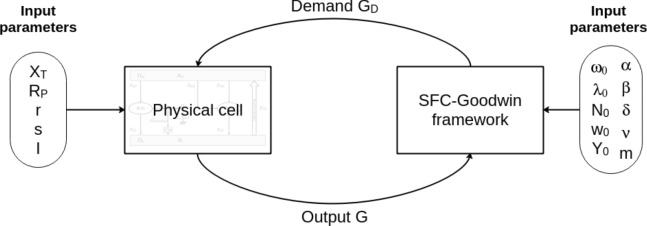
Physical sheet into a SFC-Goodwin framework.

### Connecting the bio- and the anthropo-spheres

**Figure 6:**
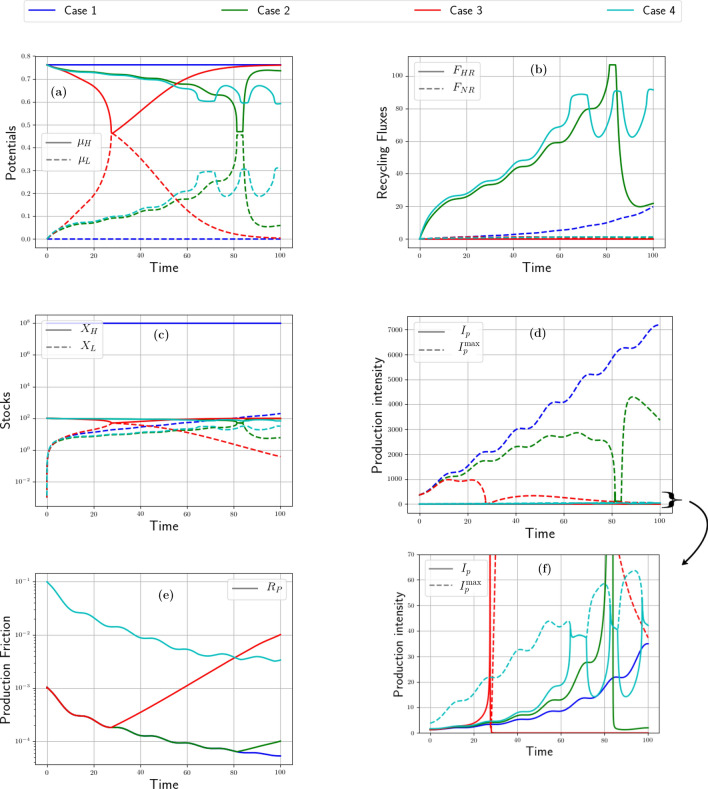
Macroeconomic simulations: resource outputs. From left to right, (**a**) Time evolution of high and low potentials ($\mu _H$ and $\mu _L$). (**b**) Time evolution recycling fluxes ($F_{HR}$ and $F_{NR}$). (**c**) Time evolution of stocks. (**d**) and (**f**) Time evolution of production intensity. (**e**) Time evolution of production friction. See Table 8 for color code.

Figure 5 depicts the interplay between the physical sheet (described in “Conversion engine in an economic context” section) and the economic framework of “The anthroposphere” section. Starting from *Y*(*t*) at time $t\ge 0$, the demand is $G_D(t):=Y(t)$. The physical sheet receives this demand from the anthroposphere and delivers an output, *G*(*t*), which depends upon the initial quantity of (natural) resources, friction, and the recycling rate. In the same way as in “Resource case studies” section, *J* is given by Eq. (31). Eventually, $G_D$ evolves according to (45). The level of friction is a decreasing function of capital.

46 $$\begin{aligned} R=\,\, & {} \frac{ R_{{{0}}}}{ K } + 4.0 \cdot 10^{-5}, \text { with } R_0>0. \end{aligned}$$

Approximating $R\simeq R_{0}/K$, and combining (38) and (40) yields

$$\overset{\varvec{.}}{S}=\frac{K}{\mu _L} \, \Bigl (\frac{R_{0} \, \rho ^2}{(\Delta \mu )^2 \, \nu ^2} + \delta \Bigr ).$$

Hence, even absent depreciation ($\delta =0$), capital accumulation *cannot* operate without bolstering entropy production. To be noticed is also the fact that entropy production is an increasing function of capital productivity, $1/\nu$. Thus, additional disorder in our habitat is the inexorable thermodynamical cost of more capital or even, simply, capital-enhancing technological progress.

The initial values presented in Table 7 come from^51^, where they are based on the reconstruction of a stylized world economy during the 2010 decade. The purpose of our simulation is to analyze the effects of finite resources, recycling intensity, and friction in a simple SFC dynamics of the Goodwin type. At variance with “Resource case studies” section, this time, investment is explicitly taken into account.

**Figure 7:**
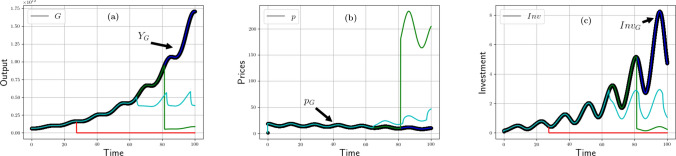
Macroeconomic simulations: economics outputs. From left to right. (**a**) Time evolution of production of output, (**b**) Time evolution of price and (**c**) time evolution of investment. See Table 8 for color code.

The baseline scenario represents a frictionless pathway absent any constraint on resources, hence without recycling either. In this scenario, output unsurprisingly grows over time following an oscillating exponential curve in line with what was observed, say, during the Thirty Glorious years in most countries of the First World. Since resources are deemed infinite, all extensive economic quantities rise over time, and the economy expands without limit. Long-run real growth is driven, as usually, by technological progress and demographic evolution whereas, after one century and a half, or so, the world economy converges to the (Solovian) balanced path stationary state. Results of this benchmark scenario are plotted with a black curve in Fig. 7a–c. A crucial question is how sensitive these results are with respect to the constraints implied by our physical sheet, and to recycling. Based on “Resource case studies” section, the following parameters vary across our next scenarios: $X_T$, *R*, recycling intensity (for simplicity, we have neglected the additional, frictional term in (39) due to capital depreciation: being linear, it is negligible in front of the quadratic term. Reckoning with it would only accelerate the pinching of the potential’s difference, albeit imperceptibly at our level of macro-dynamics writ large.)

Four classes of scenarios are plotted in Figs. 6 and 7. Table 8 wraps up the varying parameters of each scenario. As said, the first narrative (blue curve) considers an unbounded quantity of primary resources, and serves as a benchmark. The second scenario (green curve) considers, by contrast, a relatively “small” initial $X_T$. The third (red curve) and the fourth (cyan curve) ones are variations of the second. Scenario 3 (red curve) runs without any forced recycling ($n_R = 0$). Scenario 4 involves an extremely severe friction coefficient *R*. As in “Resource case studies” section, the metabolism is always supposed to exert at $J_{R}^\text {max}$, so that there are no inventories in $X_S$. The demand for resource is driven by the dynamics of “real” gdp (see Eq.  (45)), and follows the black curve of our graphs until it gets rationed.

In scenario 1 (blue curve), potentials remain constant over time and their difference is maximal throughout. This owes to the fact that, since the quantity of resources is infinitely large, the impact of human consumption remains negligible. As a consequence, resource extraction is able to fulfill the needs of production embodied by the economic dynamics. Natural recycling is relatively intense because of the depth of $X_H$. On the other hand, forced recycling turns out to be useless and, in effect, it never takes place (see Fig. 6b). Investment and inflation (Fig. 7b and c) are identical to the “pure” Goodwin case. Finally, *R* decreases courtesy of restless capital accumulation (Fig. 6e). Entropy production is enormous but has no effect, whatsoever, on the economy which evolves in an alleged world of infinite resources.

In the finite world of scenario 2 (green curve), the metabolism is able to duplicate the benchmark production path for a while but, at a certain point, production suddenly decreases and potentials are strongly pinched: the stock of initial resources has been consumed, and it can no longer be refilled in the right proportions as forced recycling is already saturated. The suddenness of this shutdown could be reminiscent of the “Seneca cliff” phenomenon analyzed by Bardi^111^. Being driven by current profits, investment (Fig. 7c) follows the production highs and lows. At the foot of the Seneca cliff, however, potentials begin to evolve thanks to the wage share, $\omega$, rising again. Indeed, the decline of profits is fast enough (in comparison to that of gdp) for wages to get a larger fraction of income back. Because of Say’s law, this results in a gradual reduction in the demand for raw materials—which allows the metabolism to “recover”. Meanwhile, natural and forced recyclings replenish the resource more easily because extraction is moderate. Eventually, the metabolism no longer operates at maximal intensity (see Fig. 6d) but it survives. As a result of the drop in production, inflation surges abruptly (see Fig. 7b), investment falls down (see Fig. 7*c*), and the state of the production system degrades more rapidly (see Fig. 6e). This might look like the decaying post-ecological catastrophe world depicted by numerous contemporary dystopias.

Scenario 3 illustrates a case where there is no artificial recycling: the resource is finite and renewed only through natural recycling. Production follows the benchmark path until resources are exhausted—at which point it collapses. Potentials and stocks follow distinct developments: the high potential climbs fairly quickly, while the stock of initial resources regenerates ploddingly (see Fig. 6c). As the resource remains fragile, production remains modest (see Fig. 7a) since it can only survive by adjusting to the slowness of natural recycling. Since production has dropped, investment did as well, and the state of infrastructures deteriorates more rapidly. This might be a scenario epitomizing the return to nature touted by some activists.

In scenario 4, the friction term, *R*, exhibits the maximal value compatible with a sustainable trajectory. In early times, production follows the benchmark path but then, its upward trend vanishes because of the pinch-off of potentials. Contrary to scenario 2, however, no break down affects the economy: it rather converges towards a limit-cycle where potentials oscillate because of the metabolizing intensity which swings in the lower vicinity of $J^\text {max}$. Indeed, the threshold $J^\text {max}$ heavily depends on the potentials and on the friction rate, which itself depends on installed capital. Investment being agitated by swaying profits, the level capital fluctuates, so that *R*, then $J^\text {max}$ and flows, stocks and potentials all swing in cadence. Prices rise gradually as a result of the successive veers in production.

We are now able to understand how the social conflict embodied within the prey-predator feature of the economic dynamics interacts with resource scarcity. Indeed, as scenarios 2 to 4 illustrate, the level of the wage share, $\omega$, and the dynamics of inflation are key elements for the sustainability of the economy. With very few exceptions, the uniform trend of wealthy countries in the past 3 decades witnessed a decrease of the wage share (hence, an increase of the profit share) and, up to 2021, a reduction of inflation (and price volatility). These two characteristics lead to higher real growth (even though growth rates in the past 30 years have been low in general, except in Asia) but also to a more rapid exhaustion of natural resources and, therefore, a more imminent upheaval of the economy. The higher the profit share and the lower is inflation, the more intensive must be forced recycling in order to prevent potentials from pinching. Since, however, recycling *also* requires high-quality energy, the recycling flux is bounded from above. This upper-bound yields a lower bound on the wage share and inflation rates. As a consequence, contrary to the current economic wisdom, less inequality (in the form of a higher wage share) and more inflation are both needed in order to enhance the ecological sustainability of an economy. These days, central banks aim at fighting against inflation by raising their leading interest rate. As, e.g., in^51^, this could be accounted for by introducing debts and the endogenous setting of a short-run interest rate according to a Taylor rule. One would then witness that increasing the interest rate can indeed succeed in taming inflation but at the cost of possibly fueling an overhang of debts which can push the economy towards an internal debt-deflationary trap, independently of resource scarcity—presumably leading to a thermo-economic scenario close to scenario 3 above.

## Conclusion: slow economy and strong sustainability

The aim of this paper has been to propose a coherent formalism of the interactions between the out-of-equilibrium thermodynamics of our degraded environment and the macrodynamics of the economic sphere. Three main features are embedded into our model: (*i*) the quantity of resources (energy and matter) is preserved throughout, as tracked by the reservoirs of initial resources and wastes together with the buffer zone of inventories and installed capital; (*ii*)  resources being available in finite quantities, only recycling allows an economy to enjoy a perennial fate; (*iii*)  in much the same way as any dissipative structure, an economy needs to heed the inexorable degradation of energy and matter; hence, recycling requires upgrading the quality of wastes, which, in turn, engages energy.

Our modeling strategy consists in building a unitary sheet for each type of resource, based on a thermodynamic conversion machine structure. Our three-sphere model—one production sphere and two recycling (natural and forced) zones—highlights how the economic metabolism uses resources to produce goods/services and to recycle waste products. Production and forced recycling vary according to the metabolizing intensity. Next, we connect this thermodynamic sheet with an economic set-up of the SFC type, fleshed out with a Goodwin-like non-linear dynamics. The intensity which maximizes short-term production (hence, profit, capital return, etc.) rapidly depletes the resource, so that the metabolism is physically constrained to quickly evolve from high to lessened intensity, while wastes accumulate. Conversely, a moderate intensity leaves time to the resource to renew itself and ultimately produces more useful work in the long-run than an initially strong operating intensity. This strategy allows a moderate but sustainable energy dissipation and material degradation. The choice of the optimal, operating intensity in general goes far beyond the simple search for optimal efficiency or maximal production, and usually requires to significantly slow down the economy. Therefore our conclusion definitely departs from the standard growth imperative but does not end up either with a plea in favor of de-growth. We indeed confirm the pessimistic conclusions already obtained with, e.g., the integrated model medeas (see^112^): “the continuation of current trends will derive in biophysical scarcities and impacts which will [ultimately derive in a] global crisis, leading to the collapse of our modern civilization”. Our standpoint, however, is more general in the sense that it does not depend upon the nitty-gritty of a full-blown iam such as medeas. Moreover, we provide a desirable alternative to collapse scenarios, based on slowing down the metabolising intensity of our economic systems. Our approach therefore provides the scientific ground for a *post-growth* perspective where, what matters, is no longer to accelerate growth and bolster profits while maintaining inflation at bay, but to slow down production, reduce inequalities by increasing wages and tolerate enough inflation for recycling to be able to save the economy from a medium-run global collapse.

The second important parameter is the friction which characterizes the economic metabolism. For two economies considered at their maximal production, a system in “good shape” will produce efficiently, i.e., with a good ratio of anabolic useful work over energy dissipation and matter degradation. However, its major drawback will be to consume more resources and to exude more wastes, if only through anthropogenic heat flux and tangible capital depreciation (1st principle). A system in worse condition will consume a lower flow of E &M, will reject less trashes, and will leave time for the resource to regenerate. As a consequence, and contrary to the main lesson drawn in economics from “Coase theorem” (see^113^), frictions are not necessarily a bad thing—even though they jeopardize economic efficiency—as they also foster sustainability by slowing down the economy. This does not imply that progress in energy efficiency is unwelcome. Notwithstanding Jevons’ paradox, higher efficiency should cause less total energy usage and thus less pollution.

As for capital accumulation, it turns out to have ambivalent consequences: even when it is assumed to reduce the internal friction of the economic engine, it can only do so at the expense of adding a linear fictional term which speeds up filling the waste sink (see (39)) and at the cost of fostering entropy production anyway. In a regionalized version of the present model (which is left for further research), the last remark could lay the ground for a two-speed development of the world economy that would remain mindful of entropy reduction: whereas humanity’s power usage rose by $\sim$2% annually until recently, one could imagine that oecd countries (whose demography is projected to stagnate, at best) would increase renewable energy at 1% annually while non-oecd countries would do so at $\sim$5% annually. Both regions would use this temporary energy bonanza to build the (green) infrastructures needed for mitigation and adaptation purposes. After zero net carbon emission would be achieved in most countries around mid-21st century, they would both continue generating more energy at most at 1% annually while the world population would plateau around $\sim 10$ bn. Residual growth in energy usage would be exclusively dedicated to capital maintenance and artificial recycling, and would be expected to gradually vanish towards the close of this century. According to^76^, a $+3\;^{\circ }$C rise of average temperature of Earth due to heat waste (on top of GHG-fueled global warming) would not occur before at least 3 centuries. On the other hand, such a twofold scenario might prevent non-oecd countries from following a path similar to scenario 3 while oecd countries would try to slow down so as to adjust by design—and not by disaster—to the post-catastrophe dynamics of scenario 2.

A number of extensions would not impair these tentative conclusions: endogenous and/or stochastic labor productivity, Putty-Clay capital structure. By contrast, some other extensions deserve further research as they would modify the phase space of the overall dynamics: introducing an endogenous population might lead to limit-cycles with an oscillating population which may or may not reinforce the limit-cycle of scenario 4.

Our sheet structure makes it possible to complexify the picture, by introducing as many sheets as required. On the other hand, our thermodynamical modeling of resources is independent of the macroeconomic model used and can be easily coupled with other alternative models.

This paper paves the road to a number of areas of research. Let us mention a few of them. First, an immediate question arises: is it possible, within the setting of this paper, to identify the basal point of the (obviously living) anthroposphere? Second, throughout the paper, we treated the metabolizing intensity as an exogenously given parameter: a next step should consist in turning it into an endogenous variable, driven by the internal forces of the economic metabolism (within the constraints imposed by the biosphere), even absent Say’s law. Third, the public sector is deliberately absent from our model as investigating its action would have gone beyond the scope of this paper. A number of policy tools could then be examined: taxes, subsidies, public investment, monetary policy, legal reforms that would change, e.g., the shape of the short-run Phillips curve, etc. The most prominent one, in our view, would be a tax on E &M that would allow to lighten the weight of taxes on labor and provide a strong incentive for substituting labour to material capital. Considering the coupling of several sheets, including one sheet for fossil fuels and another one for renewable energies, would enable to examine the impact of a carbon tax as in^51^ and enhancing subsidies for renewable energies. Fourth, for the sake of simplicity, we assumed that friction is a simple inverse function of the stock of capital. This assumption, admittedly, deserves more investigation.

### Supplementary Information

Supplementary Information.

## Data Availability

The EcoDyco code^114^, used during the current study is available in the repository: https://github.com/dyco-uparis/EcoDyco. The datasets generated and analysed during the current study are available using EcoDyco and parameters associated with the various cases.
