# Supplementary material for: Macroeconomic dynamics in a finite world based on thermodynamic potential
Source: Sci Rep. 2023 Oct 21;13:18020. doi: 10.1038/s41598-023-44699-y (PMC10590417; doi:10.1038/s41598-023-44699-y)
Supplement: Supplementary file 1 — Supplementary Information. [file 41598_2023_44699_MOESM1_ESM.pdf]

## Additional information

### List of variables

| Variable | Signification | Variable | Signification |
|---|---|---|---|
| $X_H$ | Stock of primary resource | $F_{RIn}$ | Flow of resource used to recycle |
| $X_L$ | Stock of waste | $F_{NR}$ | Flow of natural recycling |
| $X_T$ | Total stock | $R_P$ | Production viscosity |
| $X_S$ | Buffer stock | $R_R$ | Recycling viscosity |
| $\mu_H$ | Potential of primary resource | $\mathscr{J}$ | Global Intensity |
| $\mu_L$ | Potential of waste | $J$ | Production intensity |
| $F_{HP}$ | Flow of primary resource | $J^{\max}$ | Maximal intensity of production |
| $F_{LP}$ | Flow of waste | $\tau$ | Time constant of intensity of production |
| $G$ | Final goods | | |
| $G_S$ | Buffer flow | $J_R$ | Recycling intensity |
| $G_D$ | Demand of goods | $J_R^{\max}$ | Maximal intensity of recycling |
| $G_D^{\text{satisfied}}$ | (or $G_D^S$) Satisfied demand | $J_P$ | Production intensity |
| $F_{HR}$ | Flow of recycled resource | $n_P$ | Production intensity modulation coefficient |
| $F_{LR}$ | Flow of waste used in recycling | | |
| | | $n_R$ | Recycling intensity modulation coefficient |

**Table 9.** Variables summary used in this work

## 1 Multi-resources case study

We present here an illustration of a multi-resources case study. All the material necessary to run the following example is available online[114] as a free software, and accompanied by a comprehensive manual on the use and modification of the software. We will refer to this software as " the model EcoDyco ", or shorter EcoDyco in the following.

### 1.1 Parametrization

We propose to illustrate the functioning of EcoDyco by a case study based on the following resource configuration:

- Four resources are used, as shown figure 8. To ease the reading, we summarized the variables in table 9. Some are stock resources, some are flow resources.
  - Two are material; copper and wood, which are both stock resources
  - Two are energies; oil and solar energy, which are respectively stock and flux energies
- Three goods can be produced, the quantity produced depends on demand and production capacity. For each good there is a specific " recipe " that can contain energy and/or material
  - *Good 0*; can be obtained with 1 unit of copper and 2 units of energy,
  - *Good 1*; can be obtained with 1 unit of copper and 2 units of wood and 3 units of energy
  - *Good 3*; (copper recycling) can be obtained with 1 unit of energy

In addition a target energy mix is proposed including 90% of solar energy and 10% of oil. The simulation will try to full-filled this condition. If the condition is not full-filled then the code automatically switch to the most appropriate mix.

We reproduce below the contains of a parametrization file for a given sheet of a stock resource, here the copper's one. All the definitions of the parameters can be found in the online tutorial.

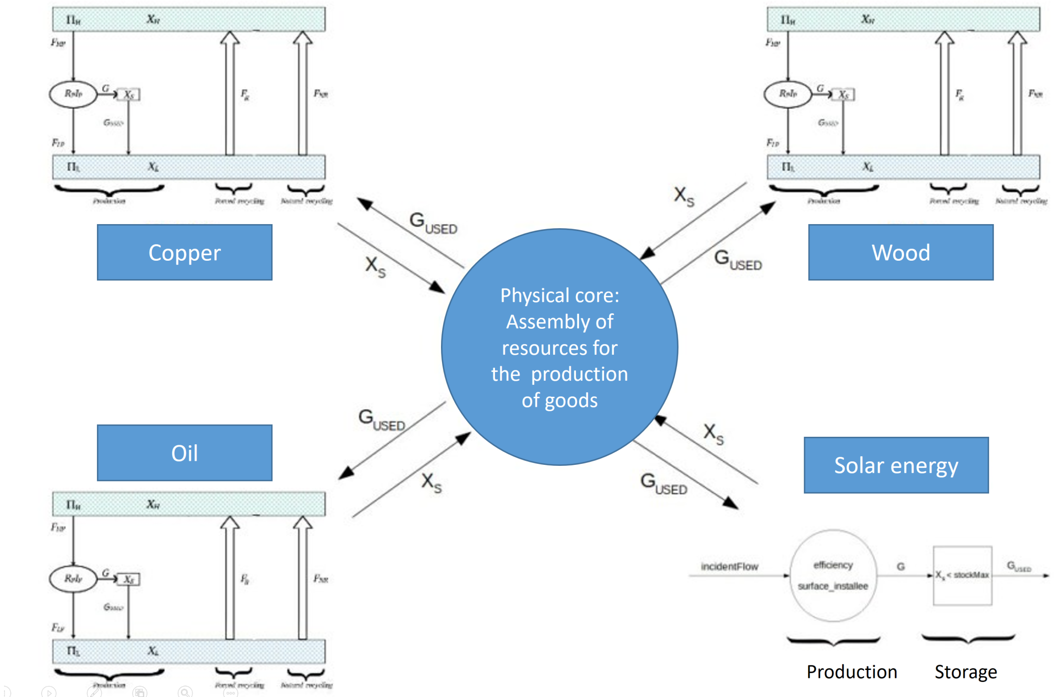


**Figure 8.** Schematic representation of the physical sphere composed of four sheets—in this example Oil, Copper, Wood are stock resources and Solar Energy is flux resource—connected to the economical sphere thanks to production of goods demand. Variables are summarized in table 9.

```
type : stock
name : copper
total stock : 500000
Xh_init : 500000
Xl_init : 0
Rp0 : 0.003
K0 : 1
recyclingEnergyFlux : 1
isEnergy : False
r : 0
to : 9
xc : 2.8e-5
x0 : 5.8e-3
```

In addition, we also report the contain of the flux sheet of the solar power production used in this modelling

```
type : flow
name : solar energy
incidentFlow : 1e10
eff_init : 0.15
installed_surface : 1e-9
isEnergy : True
stockMax_init : 50
```

The physical world is defined by a specific sheet named **world.txt**. The recipe matrix, which ensures the distribution of the different resources for each production of goods, are defined within this file.

The economic engine is set in **main.py** file. We provide as an example two economic engines, based respectively on the descriptions of Solow and Goodwin production function. In this example, the production function is of Solow-type. Parameterization is available in a dedicated files that can be modified by the user. Alternatively, another economic engine corresponding to the production function the user wishes to implement can be proposed. The production function is the only interface between the physical and economic spheres. Knowing that, all the physical parameters of each sheet are accessible at each time step of the simulation, the user can use them to decide the economic choices he wants to implement according to the state of the resources. As an illustration in the proposed example, a target energy mix is initially defined (composed of 90% solar and 10% oil) which may or may not be achieved depending on the resources.

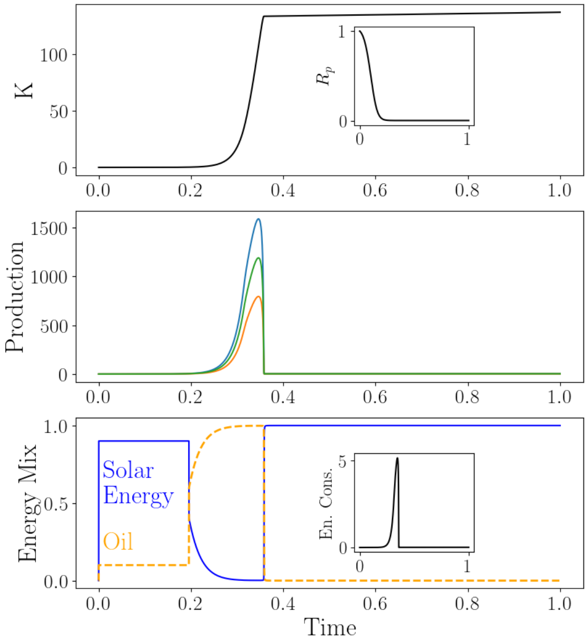


**Figure 9.** Output selection from the simulation conducted with four resources—composed of energy and matter—and Solow economics production function, see the text for details. All quantities are shown in arbitrary units, in function of a normalized time. From top to bottom: *i*-Capital variation. Inset shows $R_p$ dissipation parameter of the wood resource. $R_p = 1$ corresponds to 100% of its initial value. *ii*-Production of goods 0, 1 and 3 as defined in main text. *iii*-Energy mix, composed of oil (orange dashed thick line) and solar power (blue solid line). The target energy mix is 10% oil. In inset we show the corresponding total energy consumption.

## 1.2 Results

Running the simulation via the module **main.py** leads to the results shown in figure 9 for production capital and energy. All the information related to each sheet being recorded, it is then possible to follow the coupled evolution of their parameters, for example see in figure 10 $\Delta\Pi$ for stock sheets (*Copper*, *oil* and *Wood*) and the waste fraction (not shown).

In Figure 9, it is shown, as expected for a Solow-type production function, that capital and production of the three goods follow exponential laws up to approximately 30% of the duration of the simulation. The goods are produced in unequal quantities, corresponding to recipes and demand. Over this period, the investment allowed by the growth of capital decreases the resistance $R_p$ of each of the sheets, as shown for *Wood* as an example. The decrease of $R_p$ allows for the growth of resources production in agreement with the exponential growth production of goods. This eventually depletes the stocks until they are exhausted and the potential difference collapses simultaneously, as can be seen in figure 10, for *Wood*, *Copper* and *Oil*. The physical sphere is then no longer able to provide the resource for the economic sphere and the production of goods is almost interrupted, leaving capital to grow linearly.

The target energy mix, Figure 9 with 90% solar energy, is respected during the initial high growth period. As the growth of production is faster than the capacity to install new capture surfaces, see figure 10, *Oil* is then the only energy source able to provide the energy needed to produce the goods demanded by the economy thanks to its available stock. This constant *Oil* stock allows for the exponential growth phase. After the collapse of the production of goods, thanks to the surfaces already installed and because of the depletion of the *Oil* resource, the energy mix becomes composed almost entirely of solar energy. It is interesting to note, as shown in inset of the figure, that the total energy consumption collapsed with the production of goods.

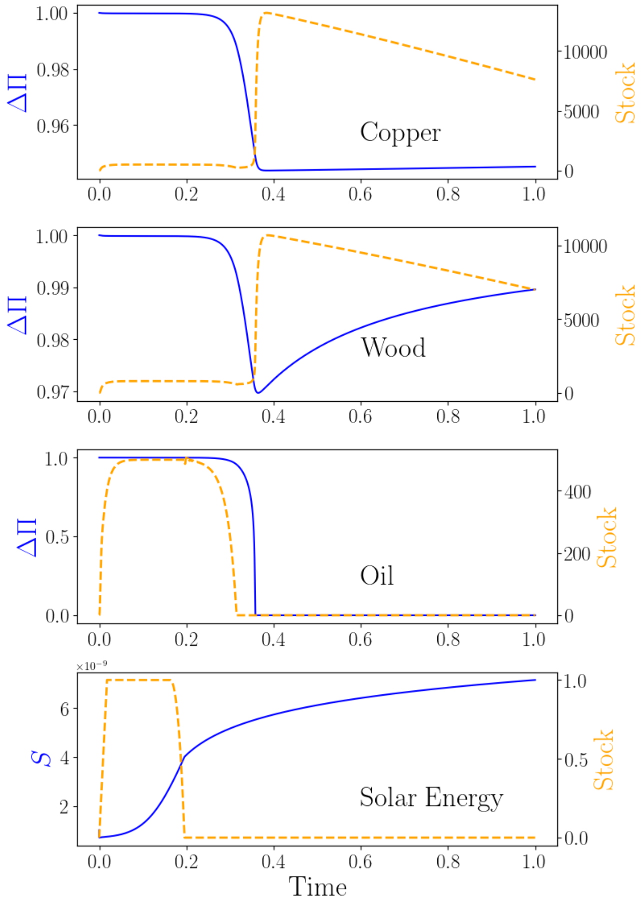


**Figure 10.** Coupled time evolution of the four resource potentials and buffer stock ($X_S$), see figure 8, involved in the simulation. See the main text for details of the simulation. We show from top to bottom: *Copper*, *Wood* and *Oil* which are stock resources and *Solar Energy* which is flux resource. For stock resources (resp. flux resource), the thin solid blue line is the potential difference $\Delta\Pi$ (resp. the installed surface) and the thick dashed orange line is buffer stock. Time is normalized. Units are arbitrary.

Figure 10 shows the comparative evolution of *Copper*, *Wood* and *Oil* sheets. Buffer stocks ($X_S$, orange lines) are generated for all resources as soon as the simulation begins. It can be seen that the exponential growth in demand generates an almost synchronised collapse of $\Delta\Pi$ (blue lines). It is *Oil* stock ($X_H$ not shown) that, in this example, is depleted first. Once the stock is consumed, the sheet is no longer able to supply energy and no longer shows any flow. This resource is no longer used in the simulation. In the case of *Wood* and *Copper* a large buffer stock ($X_S$) is formed very quickly at the time of the production collapse, the depletion of their stocks being slightly later than for *Oil*. In the continuation of the simulation we note that the recycling, natural in the case of *Wood* and forced in the case of *Copper*, makes it possible to go up $\Delta\Pi$ for both resources and thus to maintain—a very reduced but non zero—production until the end of the simulation. This production allows for the linear growth of capital observed in figure 9.
